# Sustained meningeal lymphatic vessel atrophy or expansion does not alter Alzheimer’s disease-related amyloid pathology

**DOI:** 10.1038/s44161-024-00445-9

**Published:** 2024-03-15

**Authors:** Salli Antila, Dmitri Chilov, Harri Nurmi, Zhilin Li, Anni Näsi, Maria Gotkiewicz, Valeriia Sitnikova, Henna Jäntti, Natalia Acosta, Hennariikka Koivisto, Jonathan Ray, Meike Hedwig Keuters, Ibrahim Sultan, Flavia Scoyni, Davide Trevisan, Sara Wojciechowski, Mika Kaakinen, Lenka Dvořáková, Abhishek Singh, Jari Jukkola, Nea Korvenlaita, Lauri Eklund, Jari Koistinaho, Sinem Karaman, Tarja Malm, Heikki Tanila, Kari Alitalo

**Affiliations:** 1 Wihuri Research Institute and Translational Cancer Medicine Program, Biomedicum Helsinki, University of Helsinki, Helsinki, Finland; 2 A.I. Virtanen Institute for Molecular Sciences, University of Eastern Finland, Kuopio, Finland; 3 Neuroscience Center, Helsinki Institute of Life Science, University of Helsinki, Helsinki, Finland; 4 Oulu Center for Cell-Matrix Research, Faculty of Biochemistry and Molecular Medicine, Biocenter Oulu, University of Oulu, Oulu, Finland

## Abstract

Discovery of meningeal lymphatic vessels (LVs) in the dura mater, also known as dural LVs (dLVs) that depend on vascular endothelial growth factor C expression, has raised interest in their possible involvement in Alzheimer’s disease (AD). Here we find that in the APdE9 and 5xFAD mouse models of AD, dural amyloid-β (Aβ) is confined to blood vessels and dLV morphology or function is not altered. The induction of sustained dLV atrophy or hyperplasia in the AD mice by blocking or overexpressing vascular endothelial growth factor C, impaired or improved, respectively, macromolecular cerebrospinal fluid (CSF) drainage to cervical lymph nodes. Yet, sustained manipulation of dLVs did not significantly alter the overall brain Aβ plaque load. Moreover, dLV atrophy did not alter the behavioral phenotypes of the AD mice, but it improved CSF-to-blood drainage. Our results indicate that sustained dLV manipulation does not affect Aβ deposition in the brain and that compensatory mechanisms promote CSF clearance.

AD is the most prevalent cause of dementia and one of the most significant healthcare challenges of the 21st century. Major neuropathological hallmarks of AD include extracellular Aβ plaques, consisting of 38–43 amino acid peptides cleaved from the amyloid precursor protein (APP) and intracellular neurofibrillary tangles, made of hyperphosphorylated tau protein^1^. Other features of AD include neuronal degeneration and death, cerebral amyloid angiopathy (CAA) and neuroinflammation^1,2^.

Aβ peptide accumulation is an early event in AD pathogenesis^3^. In cases of rare (<1%) dominantly inherited early-onset forms of AD, mutations in *APP* or presenilin (*PSEN*) 1 and 2 genes are causative of the disease^4^, whereas in common sporadic, late-onset AD, the initial cause of Aβ peptide accumulation is not known. The vascular system has been suggested to play a significant role in sporadic AD that is often linked to a simultaneous cerebrovascular disease^5,6^.

Research on the production and elimination of Aβ aggregates has provided important clues on the molecular pathogenesis of AD^4,7^. In late-onset AD, there is no strong evidence for an excess of Aβ production, suggesting problems in Aβ elimination^8,9^. The currently debated mechanisms of Aβ clearance include soluble Aβ clearance from the brain by a combination of transcellular transport through the blood–brain barrier (BBB) or the blood–CSF barrier, enzymatic degradation, uptake into microglia and astrocytes, brain interstitial fluid (ISF) bulk flow and ISF/CSF absorption into blood and LVs^10^. Also the ISF/CSF fluid and particle outflow into blood circulation and extracranial lymphatic vasculature has been suggested to occur by multiple pathways. These include direct efflux through the blood–CSF barrier and BBB^11^, perivascular pathways^12,13^, arachnoid villi/granulations in some animal species^14^, perineural pathways^14–19^ and the recently rediscovered dLVs^20–22^. The ISF/CSF outflow is also considered to be regulated by various physiological factors, including cardiovascular, respiratory and vasomotor pulsations as well as sleep state^23^. The relative contributions of these pathways and factors in physiological and pathological situations are still poorly understood^10,24^.

Impaired CSF clearance has been reported in patients with AD^25^. The function of the lymphatic clearance route in AD was emphasized by the finding of higher Aβ levels in cervical lymph nodes (cLNs) than in more peripheral lymph nodes in a mouse model of AD^26^ and in humans^27^. The interest in possible connection between AD development and LV function was stimulated by the rediscovery of dLVs^20,21^. These LVs have been reported to drain tracers of various sizes from the CSF and brain parenchyma into cLNs^20–22,28–30^. Ligation of extracranial LVs that lead to deep cervical lymph nodes (dcLNs) or verteporfin-mediated photodynamic ablation of dorsal dLVs aggravated brain Aβ accumulation in transgenic AD mouse models^31–33^. Furthermore, repeated injection of recombinant human vascular endothelial growth factor C (VEGF-C) into the cisterna magna (i.c.m.) induced growth of dLVs and decreased brain Aβ levels in one study^34^. In another study, mouse VEGF-C gene delivery i.c.m. failed to result in dural lymphangiogenesis or affect Aβ levels in the brain parenchyma or CSF^31^; however, it augmented brain Aβ plaque clearance when used in combination with an anti-Aβ antibody^32^.

Dural LV development and maintenance requires the VEGF-C–VEGFR3 growth factor/receptor signaling system, making it possible to induce dLV atrophy via administration of a soluble VEGF-C/VEGF-D trap that binds to and neutralizes VEGF-C and VEGF-D^22^. On the other hand, VEGF-C gene transfer has been shown to induce growth of new dLVs^22^. These tools have already been used in clinical trials, in which no safety concerns were reported^35^ (ClinicalTrials.gov identifier NCT02543229). Considering their translational potential, we have here analyzed how sustained manipulation of the VEGF-C/VEGF-D pathway to induce dLV growth or regression affects CSF outflow and amyloid neuropathology in two different transgenic mouse models of AD.

## Results

### Brain Aβ load is not increased in APdE9 mice lacking dLVs

**Extended Data Fig. 1:**
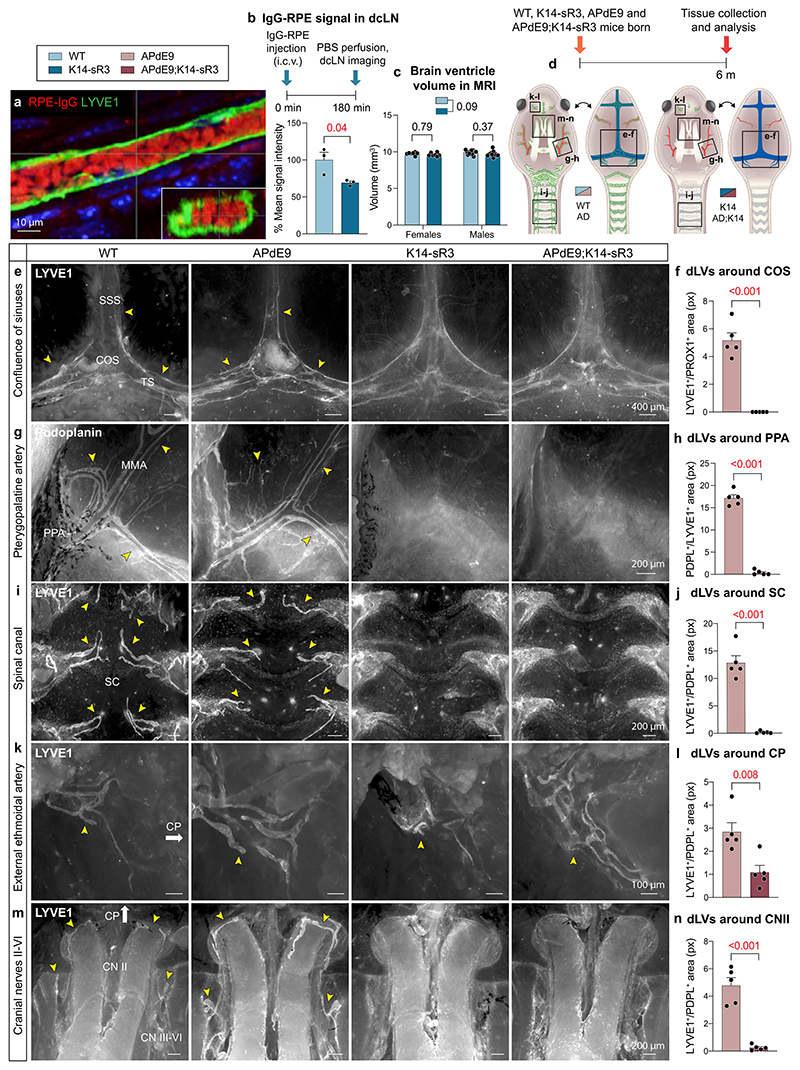
APdE9 mice lacking dLVs show impaired CSF outflow into cLNs but no change in brain ventricle volume. **a–n**, Comparison of littermate WT, APdE9, K14 and APdE9;K14 mice. Both female and male mice were used in experiments. CN, cranial nerve; COS, confluence of sinuses; CP, cribriform plate; dcLN, deep cervical lymph node; MMA, middle meningeal artery; PPA, pterygopalatine artery; SC, spinal canal; SSS, superior sagittal sinus; TS, transverse sinus. **a**, Example of ex vivo imaging of IgG-RPE (red) inside dorsal dLV (green) near COS in a WT mouse after intracranial tracer delivery. **b**, Analysis of IgG-RPE signal in dcLNs 180 minutes after i.c.v. injection (n = 3,3). IgG-RPE tracer signal values are normalized to average of WT group. LN values represent an average of both sides (left and right) amaximum one LN per side per mouse was used in quantification. **c**, Quantification of ventricle volumes imaged with MRI in 8-month-old mice (n = 7,7 for females and n = 13,13 for males) **d**, Experimental schedule for panels (**e-n**) and simplified schematic illustration of dLVs (green) attached to the basal and dorsal cranium and spinal canal after removal of the brain and spinal cord. Areas visualized in panels (**e-n**) are indicated with boxes. **e-n**, Comparison of dLVs (white) around (**e, f**) COS, (**g, h**) PPA, (**i, j**) SC, (**k, l**) external ethmoidal artery next to cribriform plate, and (**m, n**) CN II-VI region in 6-month-old mice (n = 5,5). Yellow arrowheads point to different dLV branches. White arrows point to direction of cribriform plate. Pineal gland is excised from the middle of COS in (**e**) to visualize all dLVs. Data shown are representative of at least two independent experiments using littermate mice. Datapoints shown in graphs represent individual mice. P values were calculated using (**b, f, h, j, l, n**) unpaired two-tailed *t*-test and (**c**) two-way ANOVA with Tukey’s post hoc test for multiple comparison. Data are presented as mean values ± s.e.m. Scale bars: 10 μm (**a**), 100 μm (**k**), 200 μm (**g, i, m**), and 400 μm (**e**).

To study how dLV atrophy affects the development of AD-like amyloid pathology in mice, we first used K14-VEGFR3-Ig (K14-sR3) transgenic mice^36^. In these mice, the sustained VEGF-C/VEGF-D growth factor deprivation leads to failure of dura mater LV development, without affecting dcLN size^20^. The K14-sR3 mice have impaired clearance of tracers from the brain parenchyma and CSF into dcLNs, yet they maintain a similar intracerebral pressure (ICP) as their control littermates^20^. For monitoring of CSF macromolecular clearance by dLVs, we used R-phycoerythrin (RPE)-labeled immunoglobulin (IgG), which was visualized inside dLVs and cLNs after its intraventricular (i.c.v.) delivery (Extended Data Fig. 1a,b). The results confirmed impaired drainage of IgG–RPE into the dcLNs in the K14-sR3 mice (Extended Data Fig. 1b). Using magnetic resonance imaging (MRI), we excluded possible alteration of brain ventricular volume as a pathological response to the loss of dLVs (Extended Data Fig. 1c).

We then crossed K14-sR3 mice with APdE9 mice that express human APPswe and PSEN1dE9 gene mutations from the same transgene^37^. The APdE9 mice develop the first cerebral Aβ plaques at about 4 months of age and their plaque load begins to saturate by 12 months of age^38^. Brain astrocytosis and microgliosis increase progressively with age in the APdE9 mice and their memory impairment becomes evident by 12 months of age^39,40^. To assess whether the lack of dLVs affects Aβ deposition, we analyzed the mice at the age of 6 months, when the rate of amyloid accumulation is highest and when secondary processes associated with mouse aging do not yet confound the results.

**Extended Data Fig. 2:**
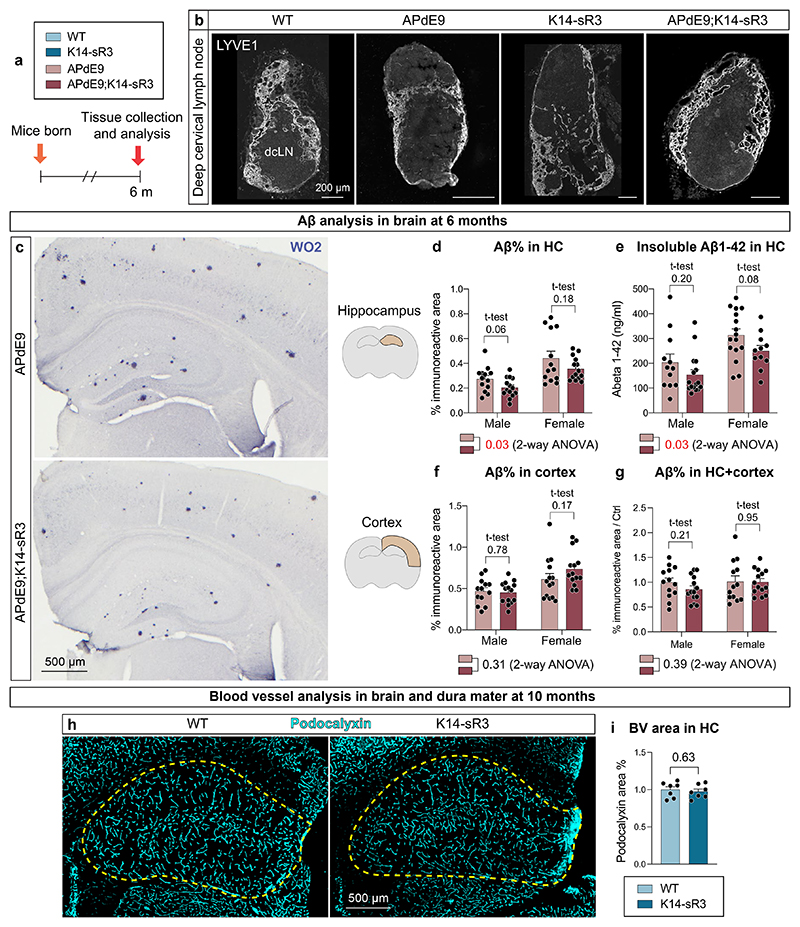
APdE9 mice lacking dLVs show no increase in overall brain Aβ load. **a-i**, Comparison of littermate WT, APdE9, K14-sR3 and APdE9;K14-sR3 mice. Both female and male mice were used in experiments. BV, blood vessel; HC, hippocampus. **a**, Experimental schedule for panels (**b-g**). **b**, Representative images of LYVE1 (white) immunostained dcLN cryosections. **c,d,f,g**, Comparison of WO2 (blue) immunoreactive area in (**c,d**) hippocampus (n = 13,14,13,14), (**c,f**) cortex above the hippocampus (n = 13,14,14,14), and (**g**) hippocampus plus cortex (n = 13,14,13,14) of male and female cohorts of APdE9 vs APdE9-K14 mice. **e**, ELISA analysis of insoluble Aβ1-42 (ng/mL) in hippocampus (n = 12,16,16,11) of male and female cohorts of APdE9 vs APdE9;K14-sR3 mice. **h-i**, Comparison of podocalyxin (cyan) staining of 10-month-old WT vs K14-sR3 female mice in hippocampus (n = 7,7). Data shown are representative of at least two independent experiments using littermate mice. Datapoints shown in graphs represent individual mice. Aβ values represent an average of 5 brain sections (210 mm apart) per mouse. Aβ values in panel (**g**) represent an average of hippocampus and cortex values that were normalized to average of APdE9-Ctrl group of every experimental set. Podocalyxin values represent an average of 2 brain sections (200 μm apart) per mouse, normalized to the average of WT group of every experimental set. *P* values were calculated using (**d-g, i**) unpaired two-tailed *t*-test and (**d-g**) two-way ANOVA with Tukey’s post hoc test for multiple comparison. Data are presented as mean values ± s.e.m. Scale bars: 200 μm (**b**), and 500 μm (**c, h**).

Wild-type (WT) and APdE9 mice had a similar pattern and location of dLVs, which were absent from the K14-sR3 and APdE9;K14-sR3 mice (Extended Data Fig. 1d–n); only some atrophic LVs were found adjacent to the external ethmoidal arteries (Extended Data Fig. 1k,l). Yet, LYVE1^+^ lymphatic endothelium in the dcLNs was retained in mice of all four genotypes (Extended Data Fig. 2b). These results demonstrate that the VEGF-C/VEGF-D trap inhibits the development of dLVs almost completely, leading to functional impairment of tracer drainage into the dcLNs.

Previous results have indicated that partial disruption of the dorsal dLVs in the 5xFAD and APP-J20 mice by verteporfin-mediated laser ablation increases Aβ load in the brain and dura mater^31,32^. To our surprise, we found that the double transgenic APdE9;K14-sR3 mice did not have higher Aβ load in the brain than the APdE9 mice (Extended Data Fig. 2c–g). Instead, the APdE9;K14-sR3 mice showed a trend for lower Aβ load in the hippocampus, as evidenced by immunohistochemistry (IHC) and enzyme-linked immunosorbent assay (ELISA) (individual *t*-tests *P* > 0.05; *P* = 0.03 in two-way analysis of variance (ANOVA); Extended Data Fig. 2c–e). The amyloid load in the cerebral cortex or the combined amyloid load in both brain areas was not altered (Extended Data Fig. 2c,f,g), suggesting that the minor decrease of plaque accumulation was restricted to the hippocampus. Hippocampal podocalyxin^+^ blood vessel (BV) coverage was similar in K14 transgenic and WT mice, confirming that the VEGF-C/VEGF-D trap does not alter blood vasculature in the hippocampus (Extended Data Fig. 2h,i). These findings indicate that the APdE9;K14-sR3 mice that lack functional dLVs do not have increased brain amyloid load.

### Brain Aβ load is not increased by dLV regression in APdE9 mice

**Fig. 1:**
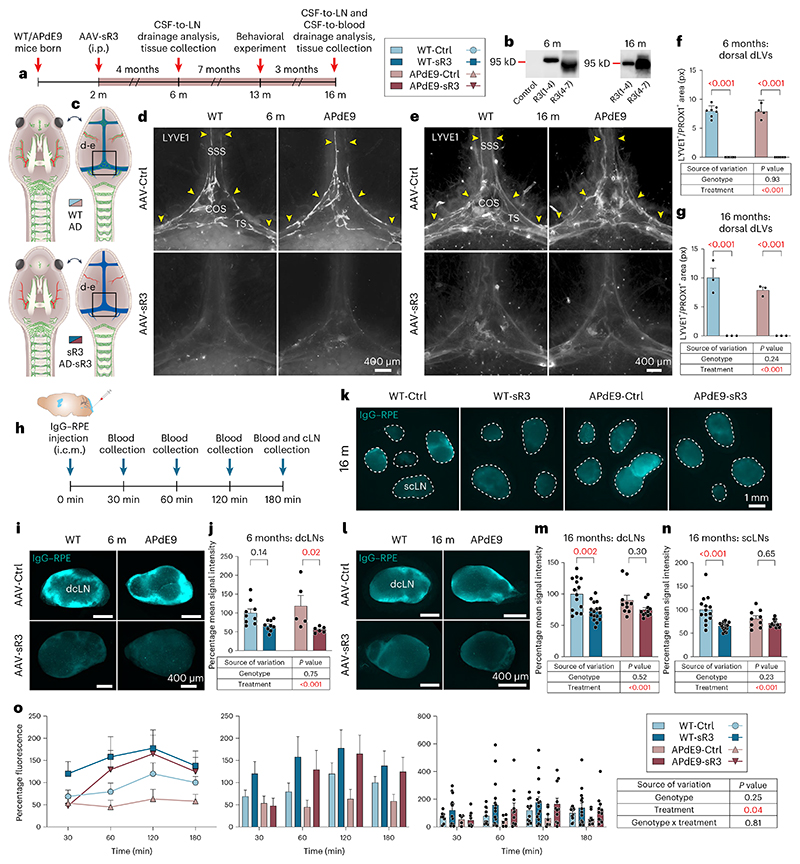
AAV-sR3 induced dLV regression in WT and APdE9 mice impairs CSF outflow into cLNs but improves CSF outflow into blood circulation. Comparison of littermate AAV-Ctrl and AAV-sR3 treated WT and APdE9 mice at 6 (female) and 16 (male) months of age. COS, confluence of sinuses; dcLN, deep cervical lymph node; scLN, superficial cervical lymph node; SSS, superior sagittal sinus; TS, transverse sinus. **a**, Schedule indicating AAV administration and experimental analysis time points. **b**, Western blot showing mVEGFR3-Ig protein in serum after AAV injection. Control sample is from a mouse with no detectable protein expression (unsuccessful injection), which was omitted from the analysis. **c**, Simplified schematic illustration of dLVs (green) attached to the basal and dorsal cranium and spinal canal. **d**–**g**, Comparison of LYVE1^+^/PROX1^+^ dLVs in the dorsal skull at 6 months (*n* = 7, 7, 4 and 6) (**d,f**) and 16 months (*n* = 3, 3, 3 and 3) (**e,g**) of age. LYVE1 staining in white (**d,e**). Yellow arrowheads point to dLV branches visible only in mice injected with AAV-Ctrl. Pineal gland was excised from the middle of COS in (**d,e**) to visualize all dLVs. **h**, The schedule of CSF drainage analysis. **i**–**n**, Comparison of IgG–RPE tracer signal in dcLNs (*n* = 9, 9, 5 and 6) of 6-month-old mice (**i,j**) and in scLNs (*n* = 14, 16, 9 and 11) and dcLNs (*n* = 14,16,10,11) (**k,n**) of 16-month-old mice 180 min after i.c.m. injection. LN values in (**j, m, n**) represent an average of both sides; maximum one LN per side per mouse was used for quantification. **o**, Kinetic analysis of IgG–RPE tracer appearance in systemic blood (saphenous vein) at 30, 60, 120 and 180 min after i.c.m. injection into 16-month-old mice (*n* = 11, 15, 9 and 11) visualized in three different ways. Data shown are representative of at least two independent experiments using littermate mice. The data points represent individual mice. LN and blood IgG–RPE tracer signal values are normalized to the average of the WT-Ctrl group of every experiment set at the 3-h time point. *P* values were calculated using two-way ANOVA (**f,g,j,m,n**) and three-way repeated measures mixed-effects model with Tukey’s post hoc test for multiple comparisons (**o**). Data are presented as mean ± s.e.m. Scale bars, 400 μm (**d,e,i,l**) and 1 mm (**k**).

Because inhibition of LV growth in developing mice is known to induce atrophy of peripheral LVs and lymph nodes (LNs)^36^ that can result in secondary developmental phenotypes, we next induced dLV regression in 2-month-old mice in which the dLV development has been completed^22^. We injected a serotype 9 adeno-associated viral (AAV) vector encoding sR3 (AAV-mVEGFR3_1–4_-Ig) or control vector (AAV-mVEGFR3_4–7_-Ig, AAV-Ctrl) intraperitoneally (i.p.) and analyzed the mice at 6 (female littermates) or 16 (male littermates) months of age, when the brain amyloid pathology is rapidly developing or fully developed, respectively (Fig. 1a). We confirmed the expression of the viral vector-produced proteins in sera from the injected mice by western blotting (Fig. 1b and Source Data Fig. 1).

**Extended Data Fig. 3:**
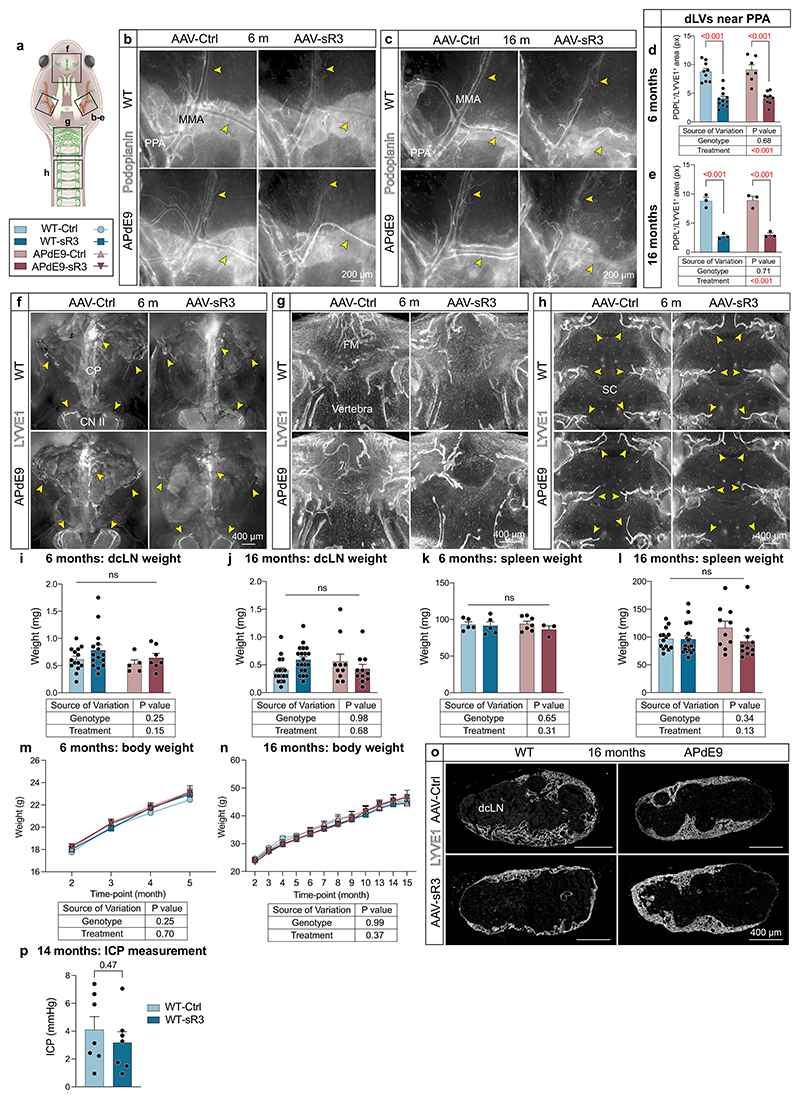
AAV-sR3 induced dLV regression in APdE9 mice does not affect LN, spleen, or body weight, or ICP. **a-p**, Comparison of littermate AAV-Ctrl and AAV-sR3 treated WT and APdE9 mice at 6 (female) and 16 (male) months of age. **a**, Simplified schematic illustration of dLVs (green) attached to the basal cranium and spinal canal after removal of the brain and spinal cord. **b–e**, Comparison of dLVs (white) in PPA region at (**b, d**) 6 months (n = 9,11,7,9), and (**c, e**) 16 months (n = 3,3,3,3) of age. **f-h**, Comparison of dLVs (white) in (**f**) CP, (**g**) foramen magnum (FM), and (**h**) SC region. **i–n**, Comparison of (**i-j**) dcLN weight at 6 (n = 14,15,5,7) and 16 (n = 17,20,10,11) months of age (**k-l**) spleen weight at 6 (n = 5,5,7,3) and 16 (n = 14,20,10,11) months of age, and (**m-n**) body weight at 6 (n = 19,19,13,11) and 16 (n = 14,20,10,11) months of age. LN weight represents an average of both sides (left and right) and maximum one dcLN per side per mouse was used in quantification. (**o**) Representative images of LYVE1 (white) staining in dcLNs. **p**, Comparison of intracranial pressure (ICP) in 14-month-old WT-Ctrl vs WT-sR3 (female mice; n = 7,7) groups 12 months after AAV injection. Yellow arrowheads point to different dLV branches. Data shown are representative of at least two independent experiments using littermate mice. Datapoints in graphs represent individual mice. *P* values were calculated with (**p**) unpaired two-tailed *t*-test, (**d, e, i-l**) two-way ANOVA, (**m**) three-way repeated measures ANOVA, and (**n**) three-way repeated measures mixed-effects model with Tukey’s post hoc test for multiple comparison in (**d, e, i–n**). Data are presented as mean values ± s.e.m. Scale bars: 200 μm (**b, e**), and 400 μm (**f-h, o**).

We have previously shown that AAV-sR3 treatment of adult mice leads to a significant and sustained dLV regression within 1 week after injection, resulting in decreased macromolecular CSF-to-dcLNs drainage^22^. In agreement with these results, our IHC analysis of AAV-sR3 treated mice at 6 or 16 months of age showed complete loss of dLVs from the dorsal dura mater and severe LV atrophy around the dural BVs and cranial nerves at the skull base (*P* < 0.001 in all, two-way ANOVA) (Fig. 1c–g and Extended Data Fig. 3a–h). The regression of basal skull dLVs was especially prominent around the pterygopalatine artery (PPA) and its branches, above the cribriform plate and around the optic nerves (Extended Data Fig. 3b–f). In contrast, dLVs around the foramen magnum and the spinal canal were affected only minimally (Extended Data Fig. 3g,h). The dLV area percentage and location were similar in the WT and APdE9 mice at both ages, indicating that the APdE9 genotype does not cause dramatic changes in dLV coverage (Fig. 1d–g and Extended Data Fig. 3b–h). Body, dcLN or spleen weights or dcLN LYVE1^+^ area coverage did not differ between genotypes or treatment groups at either age (Extended Data Fig. 3i–o).

Dural LV function was evaluated by studying the accumulation of IgG–RPE into dcLNs and superficial cLNs (scLNs) 3 h after its injection i.c.m. (Fig. 1h). In each mouse, one to two dcLNs and two to five scLNs were generally visualized on both sides of the neck. As in the K14-sR3 mice, the tracer accumulated mainly to the marginal sinus in the draining LNs (Fig. 1i,k,l). The signal from the CSF injected tracer varied between the individual LNs and some of them, especially in the scLN region, lacked signal altogether (Fig. 1i,k,l). Significantly less IgG–RPE was found in the dcLNs and scLNs in AAV-sR3 versus AAV-Ctrl-treated mice (*P* < 0.001, two-way ANOVA, about 20–40% decrease in predicted mean value) (Fig. 1i–n). In contrast, the APdE9 genotype did not significantly affect IgG–RPE drainage into cLNs at either age (Fig. 1i–n). These results confirm that AAV-sR3 causes sustained functional regression of dLVs, whereas the APdE9 genotype has no significant effect on the morphology or function of dLVs even at an advanced disease stage.

**Fig. 2:**
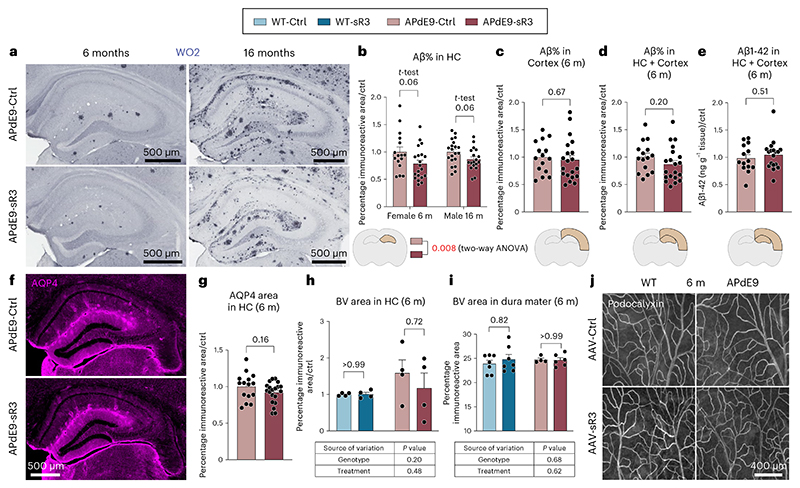
AAV-sR3 induced dLV regression in APdE9 mice does not increase the overall brain Aβ load. Comparison of littermate AAV-Ctrl- and AAV-sR3-treated WT and APdE9 mice at 6 (female) and 16 (male) months of age. BV, blood vessel; HC, hippocampus. **a,b**, Comparison of WO2 (blue) staining of hippocampus in coronal brain sections in 6-month-old (*n* = 16 and 20) and 16-month-old (*n* = 20 and 18) mice. **c,d**, Quantification of Aβ% in cortex (**c**) and HC plus cortex (**d**) in 6-month-old (*n* = 15 and 20) mice. **e**, ELISA quantification of insoluble Aβ1-42 (ng g^−1^) in the brains in 6-month-old (*n* = 14 and 18) mice. Each data point represents an average of HC and cortex values. **f,g**, Comparison of aquaporin-4 (AQP4, magenta) staining of HC in 6-month-old mice (*n* = 15 and 20). **h**–**j**, Comparison of podocalyxin (white color in **j**) staining in HC (*n* = 4, 4, 4 and 4) (**h**) and dorsal dura mater (*n* = 7,7,4,6) (**i,j**) in 6-month-old mice. Data shown are representative of at least two independent experiments using littermate mice. Data points shown in graphs represent individual mice. Aβ values represent an average of five brain sections (210 mm apart) per mouse. W02, Aβ1-42, AQP4 and brain podocalyxin values were normalized to average of the APdE9-Ctrl group of every experimental set. *P* values were calculated with unpaired two-tailed *t*-test (**b**–**e,g**) and two-way ANOVA (**b,h,i**) with Tukey’s post hoc test for multiple comparison. Data are presented as mean values ± s.e.m. Scale bars, 400 μm (**j**) and 500 μm (**a,f**).

We next quantified the amyloid plaques in the hippocampus and cortex in anti-Aβ stained brain sections. Unexpectedly, the regression of dLVs did not increase the Aβ immunoreactive area in the hippocampus, unlike in the study in which dorsal dLVs were partially destroyed by photoablation in 5xFAD and APP-J20 mice^31^. Instead, similarly as in the APdE9;K14-sR3 mice, the hippocampal Aβ immunoreactive area in the AAV-sR3 treated APdE9 mice showed a trend for decreased amyloid load (individual *t*-tests *P* = 0.06; *P* = 0.008 in two-way ANOVA) (Fig. 2a,b). The Aβ immunoreactive area in the cortex above the hippocampus, or the combined amyloid load of both brain areas, was not significantly affected (Fig. 2c,d). Human Aβ-specific ELISA did not show significant differences in the combined analyses (Fig. 2e). Quantification of BV coverage and aquaporin-4 (AQP4) expression in the perivascular astrocyte end feet facing the BBB in hippocampus was not significantly different between APdE9 mice treated with AAV-sR3 or AAV-Ctrl (Fig. 2f-h). These findings indicate that sustained impairment of dLV function by the VEGF-C/VEGF-D trap treatment does not increase the overall brain amyloid load in APdE9 mice.

**Extended Data Fig. 4:**
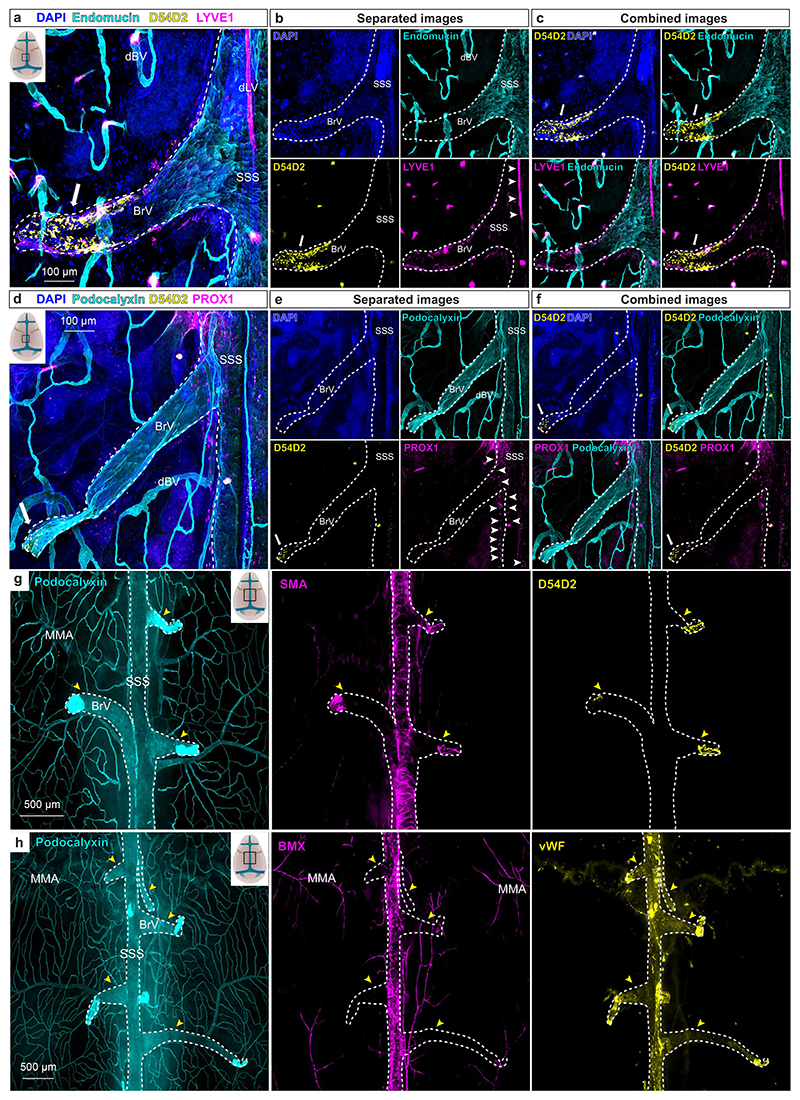
Aβ staining in dura mater is associated mainly with bridging veins. **a**-**h**, Whole-mount stainings of dorsal dura mater. BrV, bridging vein; dBV, dural blood vessel; dLV, dural lymphatic vessel. SSS and BrVs connecting to it are outlined in all panels by the white dashed line. **a**-**f**, Representative confocal images of DAPI (blue), endomucin (cyan), D54D2 (yellow), and LYVE1 (magenta) staining (**a**-**c**) and DAPI (blue), podocalyxin (cyan), D54D2 (yellow), and PROX1 (magenta) staining (**d**-**f**) in old APdE9 mouse (22-month-old non-treated female). White arrows indicate Aβ staining associated with BrV. White arrowheads indicate dLVs. **g**-**h**, Representative stereomicroscope images of podocalyxin (cyan), SMA (magenta), and D54D2 (yellow) staining in adult APdE9 mouse (9,5-month-old male) (**g**) and podocalyxin (cyan), BMX (magenta, stained with RFP), and vWF (yellow) staining in adult BmxCreER^T2^Rosa26^LSL-TdTomato^ mouse (13-month-old male) (**h**). BrVs connecting to SSS are marked with yellow arrowheads. Data shown are representative of minimum n = 3 for every staining. Scale bars: 100 μm (**a**-**f**), and 500 μm (**g, h**).

**Fig. 3:**
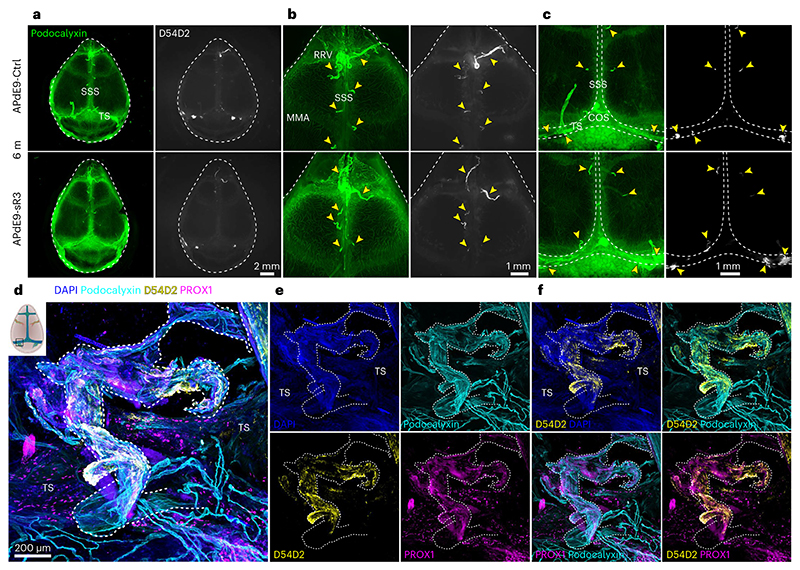
Aβ deposits in dura mater are associated with bridging veins, but not with dLVs. **a**–**c**, Representative images of podocalyxin (green) and D54D2 (white) staining in skull-associated dorsal dura in 6-month-old female APdE9 littermate mice treated with AAV-Ctrl- or AAV-sR3. Dorsal dura mater (**a**). Rostral part (**b**). Caudal part (**c**). MMA, middle meningeal artery; RRV, rostral rhinal vein. Yellow arrowheads point to areas where D54D2^+^ amyloid deposits associate with podocalyxin^+^ bridging veins that connect to large dural sinuses. Pineal gland was excised in **a,c** to visualize all blood vessels. **d**–**f**, Representative image of 4,6-diamidino-2-phenylindole (DAPI) (blue), podocalyxin (cyan), D54D2 (yellow) and PROX1 (magenta) staining in a 22-month-old non-treated female APdE9 mouse. Image with all stainings combined (**d**). Separated images (**e**). Images with two stainings combined (**f**). The dotted line marks the podocalyxin^+^ bridging vein that connects to the TS and is associated with most D54D2^+^ Aβ deposits in the TS region. Data shown are representative of at least two independent experiments using littermate mice. Scale bars, 1 mm (**b,c**), 2 mm (**a**) and 200 μm (**d**).

Next, we assessed Aβ deposition in dura mater. We found intense anti-Aβ staining in the dorsal, but not in the basal, dura mater in both AAV-sR3- and AAV-Ctrl-treated APdE9 mice. Notably, unlike in the photoablation study^31^, we detected Aβ staining also in the dura mater of AD mice without dLV regression (Fig. 3a–f and Extended Data Fig. 4a–h). The staining was mainly associated with podocalyxin^+^/vWF^+^/BMX^−^ BVs connecting brain to dura mater, namely bridging veins, that are known to drain blood from brain into the large dural venous sinuses (Fig. 3a–f and Extended Data Fig. 4a–h). The most prominent Aβ staining was associated with the bridging veins that join transverse sinuses (TSs) and with the rostral end of the superior sagittal sinus (SSS) (Fig. 3a–f).

However, Aβ staining was associated with the bridging veins all along the SSS (Fig. 3a–f and Extended Data Fig. 4a–h). In comparison to large dural venous sinuses, the bridging veins showed lower endomucin staining in endothelium and lacked SMA^+^ smooth muscle cell coating (Extended Data Fig. 4a,g). Aβ staining did not consistently colocalize with dLVs (Fig. 3d–f and Extended Data Fig. 4a–f) and staining was present in similar locations also in the dura mater of mice lacking the dorsal dLVs (Fig. 3a–c). There was no difference in the dural BV area between sR3-versus Ctrl-treated APdE9 mice (Fig. 2i,j). The association of dura mater Aβ deposits mainly with BVs, but not LVs, further suggests that the dLVs are not directly involved in draining of Aβ from the central nervous system (CNS) in the APdE9 model. Moreover, this data suggests that bridging veins connecting the brain with dura mater and/or the perivascular space surrounding them provide a route for brain-derived Aβ drainage into the meningeal layers.

### sR3 improves transfer of a CSF tracer to blood circulation

To characterize the effects of the APdE9 genotype and AAV-sR3 treatment on the overall CSF efflux into the systemic circulation, we monitored fluorescence in saphenous vein blood samples at 30, 60, 120 and 180 min after i.c.m. administration of the IgG–RPE tracer to 16-month-old mice (Fig. 1o). Even though the fluorescence levels seemed somewhat lower in APdE9 mice than in WT mice, the genotype did not significantly affect drainage of the macromolecular tracer from CSF into blood (Fig. 1o). Unexpectedly, the IgG–RPE mean signal intensity in venous blood was significantly stronger in AAV-sR3-injected than in AAV-Ctrl-injected mice (*P* = 0.04, three-way repeated measures mixed-effects model) (Fig. 1o). Overall, the signal declined in all groups after 120 min (Fig. 1o). The ICPs were not significantly different between AAV-sR3-versus AAV-Ctrl-treated WT mice after 12 months of treatment, indicating that the CSF outflow system can compensate for the loss of the dLVs (Extended Data Fig. 3p). This suggests that the improved drainage into blood circulation was not caused by elevated ICP, which is in line with our previous finding that the K14-sR3 mice and their WT littermates have similar ICP levels^20^. Thus, our results indicate that the AAV-sR3 treatment improves clearance of CSF macromolecules into systemic blood circulation, presumably in response to dLV atrophy.

### dLV regression does not affect the behavior of APdE9 mice

**Extended Data Fig. 5:**
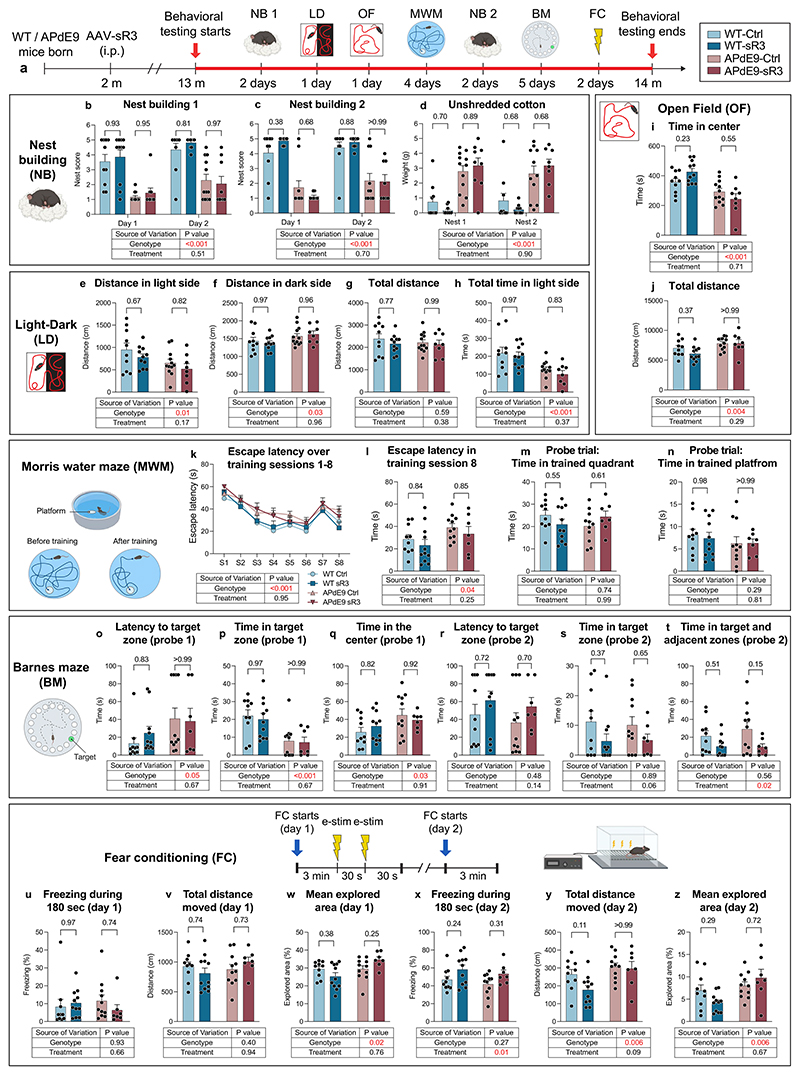
AAV-sR3 induced dLV regression causes only modest changes in the behavioral phenotype of WT and APdE9 mice. **a-z**, Comparison of behavioral results in littermate male AAV-Ctrl treated WT (n = 10), APdE9 (n = 11) and AAV-sR3 treated WT (n = 12) and APdE9 (n = 9) mice at 13-14 months of age. NB, nest building; LD, Light–dark; OF, Open field; MWM, Morris water maze; BM, Barnes maze; FC, Fear conditioning. **a**, Experimental schedule of all behavioral tests. **b-d**, NB results showing (**b**) nest scores at timepoint 1 (**c**) nest scores at timepoint 2, and (**d**) amount of unshredded cotton at the end of both time points. **e-h**, LD results showing total (**e**) distance traveled in light side (**f**) distance traveled in dark side (**g**) distance traveled, and (**h**) time spent on light side. **i-j**, OF results showing total (**i**) time spent at the center of open field arena and (**j**) distance moved in open field arena. **k-n**, MWM results showing (**k**) escape latency in eight training sessions (S1-S8; total four days with two training sessions per day). (**l**) escape latency in last training session (S8, reverse training), (**m**) total time spent in trained quadrant in probe trial, and (**n**) total time spent in trained platform in probe trial. **o-t**, BM experimental results showing (**o, r**) latency to target zone in probe trials 1-2, (**p, s**) time spent in target zone in probe trials 1-2, (**q**) time spent at the center of arena in probe trial 1, and (**t**) time spent in target and adjacent zones of the arena in probe trial 2. **u-z**, FC results at day 1 and 2 showing (**u, x**) freezing % during 180 seconds, (**v, y**) total distance moved, and (**w, z**) mean explored area %. Data shown are representative of a single experiment using littermate mice. Datapoints in graphs represent individual mice. The NB, MWM, BM and FC illustrations were created with BioRender.com. *P* values were calculated using (**e-j, l-z**) two-way ANOVA, (**b-d**) two-way repeated measures ANOVA, and (**b-d, k**) three-way repeated measures ANOVA all with Tukey’s post hoc test for multiple comparison. Data are presented as mean values ± s.e.m.

To assess the effect of sustained dLV regression on cognitive function, we performed several behavioral experiments in 13 month-old AAV-Ctrl versus AAV-sR3 treated WT and APdE9 mice, including assessment of species-specific goal-oriented behavior (nest building), spatial learning and memory (Barnes maze and Morris water maze), contextual fear conditioning (fear conditioning experiment), as well as anxiety (open field and light–dark) (Extended Data Fig. 5a). As expected, we consistently found significant differences in all behavioral results between the APdE9 versus WT mice (Extended Data Fig. 5b–z), but not between the AAV-sR3-versus AAV-Ctrl-treated mice (Extended Data Fig. 5b–z). However, the AAV-sR3-treated mice showed a trend toward improved contextual fear conditioning (Extended Data Fig. 5z) and impaired learning of the new escape hole location in the Barnes maze (Extended Data Fig. 5t). These findings indicate that the sustained dLV regression resulting from the AAV-sR3 treatment does not significantly affect the behavioral phenotype of the APdE9 mice.

### Aβ load is not affected by dLV regression in 5xFAD mice

**Fig. 4:**
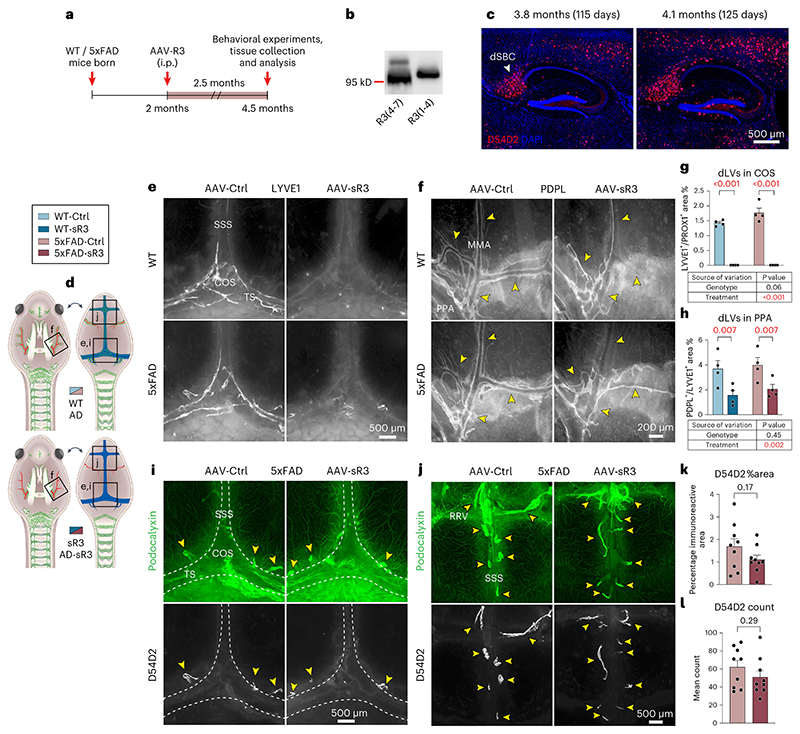
Dural Aβ load in 5xFAD mice is not affected by AAV-sR3 induced dLV regression. Comparison of littermate AAV-Ctrl- and AAV-sR3-treated WT and 5xFAD male mice at 4.5 months of age. **a**, Schedule indicating AAV administration and experimental analysis time points. **b**, Western blot showing mVEGFR3-Ig protein in serum after AAV injection. **c**, Representative D54D2 staining (red) in the HC of littermate mice with 10-d age difference. dSBC, dorsal subiculum. **d**, Simplified schematic illustration of dLVs (green) attached to the basal and dorsal cranium and spinal canal. **e-h**, Comparison of LYVE1 (white) staining in COS (*n* = 4,4,4,4) (**e,g**) and podoplanin (white) staining in PPA region (*n* = 4, 4, 4 and 4; average of left and right side) (**f,h**). Yellow arrowheads point to basal dLV branches that show robust regression after sR3 treatment. **i**–**j**, Comparison of D54D2 (white) and podocalyxin (green) staining in caudal (**i**) and rostral (**j**) dorsal dura mater. Yellow arrowheads point to areas where D54D2^+^ Aβ deposits colocalize with podocalyxin^+^ bridging veins that connect to large dural sinuses. **k-l**, Quantification of D54D2 %area and count (*n* = 9,9) in the caudal region of dorsal dura mater visualized in **i**. Data shown are representative of at least two independent experiments using littermate mice. Data points shown in graphs represent individual mice. Pineal gland was excised (**e,i**) to visualize all blood and lymphatic vessels. *P* values were calculated using two-way ANOVA with Tukey’s post hoc test for multiple comparison (**g,h**) and unpaired two-tailed *t*-test (**k,l**). Data are presented as mean ± s.e.m. Scale bars, 200 μm (**f**) and 500 μm (**e,i,j**).

AD-like amyloid pathology develops very fast in 5xFAD mice, in which the Aβ deposits appear at about 2 months of age and saturate at about 9 months of age in the cerebral cortex and develop more slowly in the hippocampus^41^. Brain astrocytosis and microgliosis in 5xFAD mice increase progressively with age, and the motor, emotional and cognitive phenotype becomes evident by 6 months of age^41,42^. Recent studies indicated that repeated photodynamic treatment of 5xFAD mice for 6–8 weeks, starting at 1.5–3.5 months of age, to ablate dorsal dLVs increases the accumulation of Aβ in the brain compared to sham-treated mice^31,32^. We used a similar schedule in our experiment, with AAV-sR3 injection at 2 months of age and analysis at 4.5 months of age, followed by verification of expression of sR3 protein in serum (Fig. 4a,b and Source Data Fig. 4). The injection and end-point analysis were conducted within a 5-d time window to avoid variation in disease phenotype within groups (Fig. 4c).

**Fig. 5:**
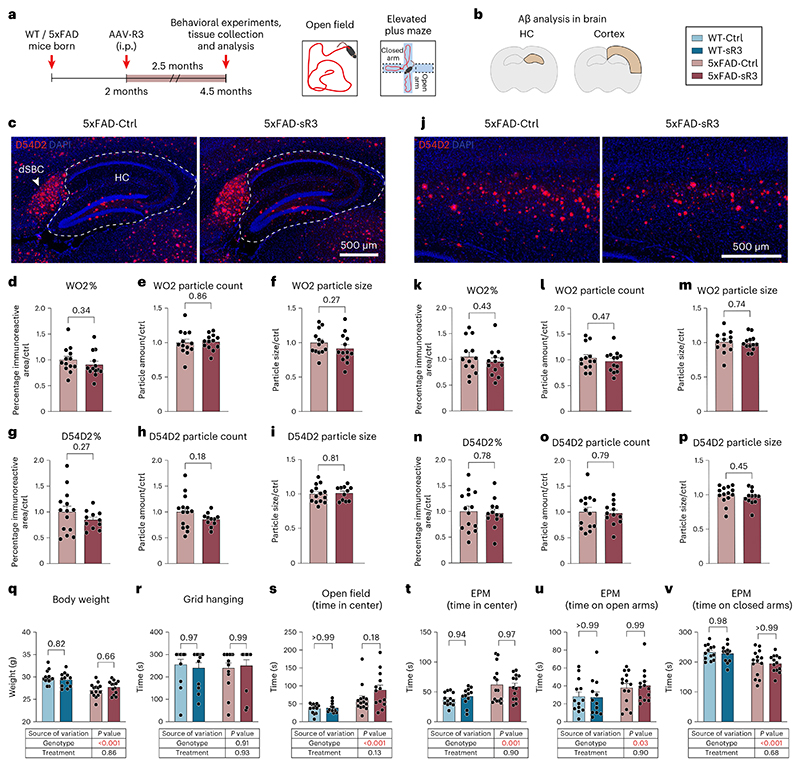
AAV-sR3 induced dLV regression does not affect brain Aβ load in 5xFAD mice. Comparison of littermate AAV-Ctrl- and AAV-sR3-treated WT and 5xFAD male mice at 4.5 months of age. **a**, Schedule indicating AAV administration and experimental analysis time points. **b**, Schematic illustration indicating brain quantification areas in panels (**c**–**p**). **c,j**, Representative images of D54D2 Aβ staining (red) in HC (**c**) and cortex (**j**) region. Example of a quantified HC area without dSBC is outlined with the white dashed line. **d**–**p**, Quantification of Aβ staining in HC (WO2; *n* = 13 and 13 and D54D2; *n* = 14 and 11) (**d**–**i**) and cortex (WO2; *n* = 13 and 13 and D54D2; *n* = 14 and 12) (**k**–**p**) region. **q**–**v**, Comparison of body weight (**q**), grid hanging (**r**), open field (**s**) and elevated plus maze (EPM) (**t**–**v**) results (*n* = 13, 12, 14 and 13). Data shown are representative of at least two independent experiments using littermate mice. Data points shown in graphs represent individual mice. Brain Aβ values represent an average of six brain sections (400 μm apart) per mouse and are normalized to average of 5xFAD-Ctrl group of every experimental set. *P* values were calculated using unpaired two-tailed *t*-test (**d**–**i,k**–**p**) and two-way ANOVA with Tukey’s post hoc test (**q**–**v**) for multiple comparisons. Data are presented as mean ± s.e.m. Scale bars, 500 μm (**c,j**).

As in the APdE9 mice, a complete regression of the dorsal dLVs (*P* < 0.001, two-way ANOVA) and a significant regression of the basal dLVs (*P* = 0.002, two-way ANOVA) were obtained also in the 5xFAD mice expressing the VEGF-C/VEGF-D trap (Fig. 4d–h). In dura mater, the dLV regression did not affect the anti-Aβ staining that was again associated with the bridging veins (Fig. 4i–l). In the brain, anti-Aβ staining using D54D2 or WO2 antibodies did not reveal significant differences in hippocampal or cortical amyloid deposits between AAV-sR3-treated versus control 5xFAD mice (Fig. 5a–p). The regression of the dLVs did not affect body weight or the tested behavioral parameters, including nest building, motor ability (grid hanging) or anxiety (open field and elevated plus maze) (Fig. 5q–v). These data indicate that AAV-sR3-induced sustained dLV regression does not affect the disease phenotype in 5xFAD mice.

### VEGF-C-induced dLV expansion does not alter Aβ load in APdE9 mice

**Extended Data Fig. 6:**
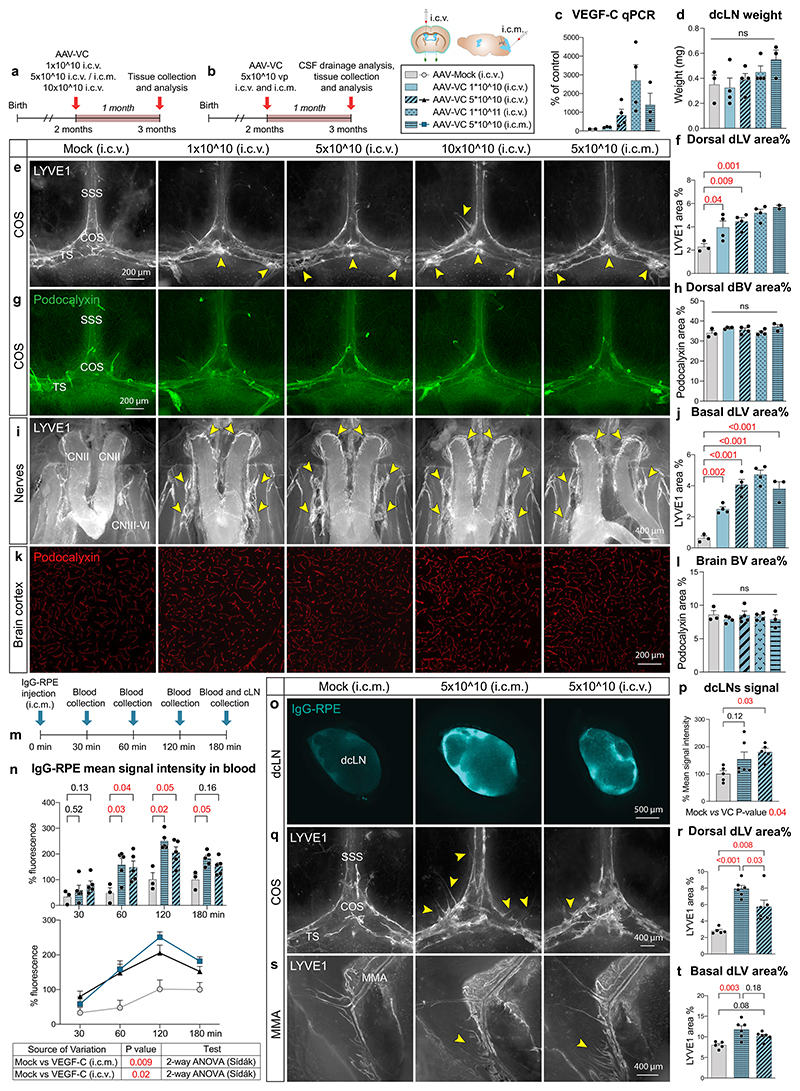
AAV-VC induced dLV expansion improves CSF outflow into cLNs and blood circulation in WT mice. **a**, Experimental schedule for panels **c-l** with male AAV-Mock (i.c.v.), AAV-VC 1*10^10^ vp (i.c.v.), AAV-VC 5*10^10^ vp (i.c.v.), AAV-VC 1*10^11^ vp (i.c.v.), and AAV-VC 5*10^10^ vp (i.c.m.) groups. **b**, Experimental schedule for panels (**m-t**) with female AAV-Mock (i.c.v.), AAV-VC 5*10^10^ vp (i.c.v.), and AAV-VC 5*10^10^ vp (i.c.m.) groups. **c**, Quantification of VEGF-C mRNA levels in brain cortex (n = 2,3,4,4,3). **d**, Quantification of dcLN weight (n = 3,4,4,4,3). **e-j**, Comparison of dura mater (**e, f**) LYVE1 area (white) in dorsal skull around COS (n = 3,4,4,4,2), (**g, h**) podocalyxin area (green) in dorsal skull (n = 3,4,4,4,32; two areas per mouse around large dural sinuses), (**i, j**) LYVE1 area (white) in basal skull around CNII-VI (n = 3,4,4,4,3). Yellow arrowheads in **e, g** point towards areas with most prominent lymphangiogenesis. **k-l**, Podocalyxin immunoreactive area (red) in brain cortex (n = 3,4,4,4,3). **m-n**, Kinetic analysis of IgG-RPE tracer appearance in systemic blood at 30, 60, 120 and 180 min after i.c.m injection (n = 3,5,5), visualized in two different ways. **o-p**, Comparison of IgG-RPE tracer signal in dcLNs at 180 min after i.c.m injection (n = 5,6,5). **q-t**, Comparison of LYVE1 (white) staining (n = 5,6,6) in (**q, r**) COS and (**s, t**) MMA region. Yellow arrowheads point towards new lymphatic sprouts. Panels (**c-l**) and (**m-t**) represent single independent experiments using littermate mice. Datapoints shown in graphs represent individual mice. LN values in (**d, p**) represent an average of both sides (left and right); maximum one LN per side per mouse was used in quantification. LN and blood IgG-RPE tracer signal values are normalized to the average of WT-Mock group of every experimental set at the 3 h timepoint. The pineal gland was excised in (**e, g, q**) to visualize blood and lymphatic vasculature. *P* values were calculated using one-way ANOVA with (**d, f, h, j, l, p**) Dunnett’s post hoc or (**r, t**) Tukey’s post hoc or (**n**) two-way repeated measures ANOVA with Dunnett’s and Sidak’s post hoc tests for multiple comparison. Data are presented as mean values ± s.e.m. Scale bars: 200 μm (**e, g, k**), 400 μm (**i, q, s**) and 500 μm (**o**).

Injection of AAV9-mVEGF-C (encoding the full-length VEGF-C, hereafter AAV-VC) i.c.v. or i.c.m. in mice induces robust lymphangiogenesis in the dorsal and basal dLVs but does not affect BBB permeability^22^. Here, we first compared the dLVs in mice injected i.c.v. or i.c.m. with 1 × 10^10^, 5 × 10^10^ or 1 × 10^11^ AAV-VC or AAV-Mock particles per mouse. After 4–5 weeks, we analyzed VEGF-C expression in the brain, CSF tracer drainage, dural LVs and BVs, and brain BVs (Extended Data Fig. 6a,b).

AAV-VC induced a trend for dose-dependent exponential increase in VEGF-C messenger RNA in the cortex (Extended Data Fig. 6c) and for more linear increase of LV area% in both the dorsal and ventral dura mater (Extended Data Fig. 6e,f,i,j). The lymphangiogenic effect of AAV-VC was evident throughout the dorsal dura (Extended Data Fig. 6e,f,q,r), most prominently around the areas where the bridging veins connect to the TSs, SSS (especially the rostral end) and confluence of sinuses (Extended Data Fig. 6e,q). In the basal dura mater, the most prominent lymphangiogenesis occurred in the rostral areas around cranial nerves II–VI and above the cribriform plate in the olfactory bulb cavity, whereas only moderate expansion of LVs was detected around the PPA/middle meningeal artery; dLVs around the foramen magnum and spinal canal were not affected (Extended Data Fig. 6i,j,s,t). In several locations, the lymphatic sprouts seemed to grow along podocalyxin^+^ BVs connecting to large dural sinuses (Extended Data Fig. 6e,g). Deep cervical LN weight or dural or brain cortical BV area percentages were not affected by AAV-VC transduction (Extended Data Fig. 6d,k,l). The i.c.m.-injected AAV-VC induced slightly stronger lymphangiogenesis in the dura mater, especially in the dorsal part, than a similar dose injected i.c.v. (Extended Data Fig. 6e,f,q,r); however, both i.c.m.- and i.c.v.-injected AAV-VC increased the overall mean IgG–RPE signal intensity in venous blood circulation versus Mock-treated mice (*P* = 0.009 and *P* = 0.02, respectively; two-way ANOVA for repeated measures; Extended Data Fig. 6n). Also, the IgG–RPE fluorescence in the dcLNs was stronger in AAV-VC injected mice (*P* = 0.04; two-way ANOVA; Extended Data Fig. 6o,p). Collectively, these results demonstrate that AAV-VC induces dose-dependent dural lymphangiogenesis, leading to improved clearance of CSF macromolecules into cLNs and blood circulation.

**Extended Data Fig. 7:**
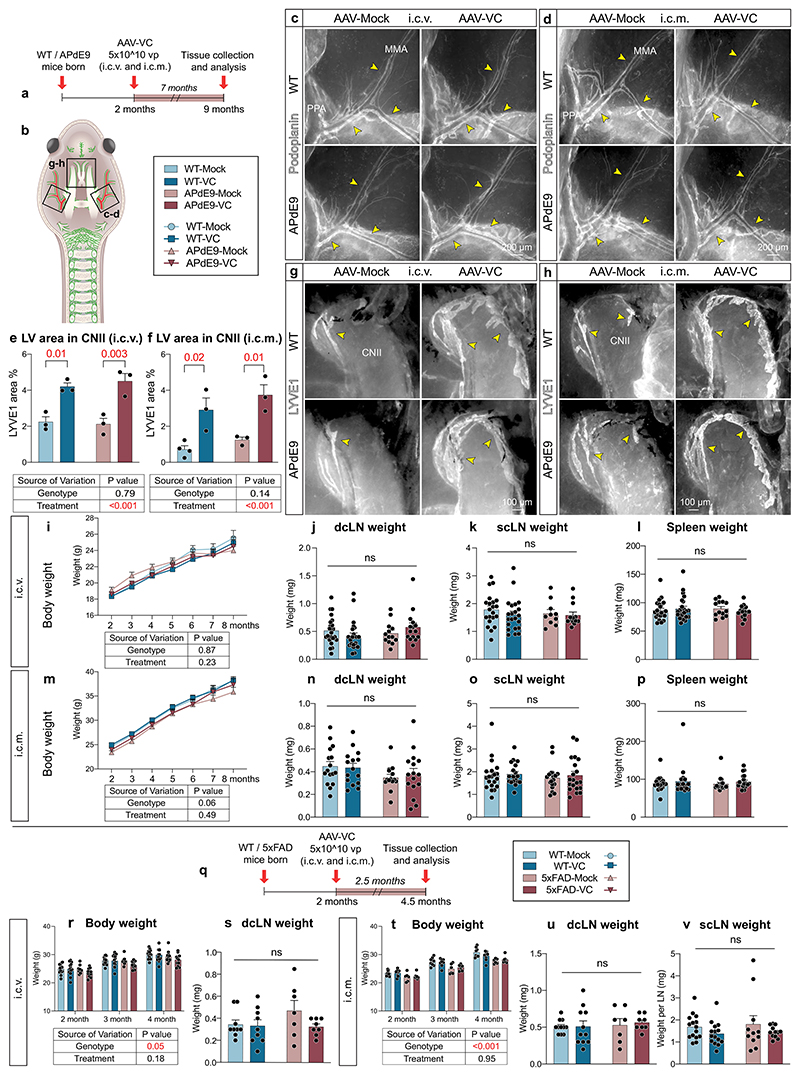
AAV-VC induced dLV expansion in APdE9 and 5xFAD mice does not affect LN, spleen, or body weight. **a**, Experimental schedule for panels **b–p**; comparison of littermate AAV-Mock and AAV-VC-treated i.c.m. (male) and i.c.v. (female) injected WT and APdE9 mice at 9 months of age. **b**, Simplified schematic illustration of dLVs (green) attached to the basal cranium and spinal canal after removal of the brain and spinal cord. **c-h**, Comparison of dLVs (white) in (**c-d**) PPA and (**e-h**) CNII region of i.c.v. (n = 3,3,3,3) and i.c.m. (n = 4,3,3,3) injected APdE9 and WT mice. **i-l**, Comparison of (**i**) body (n = 23,23,13,15), (**j**) dcLN (n = 23,23,13,15), (**k**) scLN (n = 21,22,10,13), and (**l**) spleen weight (n = 23,23,13,15) in i.c.v. injected APdE9 and WT mice. **m-p**, Comparison of (**m**) body (n = 20,19,18,20), (**n**) dcLN (n = 16,15,14,16), (**o**) scLN (n = 20,18,18,20), and (**p**) spleen weight (n = 20,19,18,20) in i.c.m. injected APdE9 and WT mice. **q**, Experimental schedule for panels **r-v**; comparison of littermate AAV-Mock and AAV-VC-treated i.c.m. (male and female) and i.c.v. (male) injected WT and 5xFAD mice at 4.5 months of age. **r-s**, Comparison of (**r**) body (n = 13,13,11,12) and (**s**) dcLN (n = 9,9,7,8) weight in i.c.v. injected 5xFAD and WT mice. **t-v**, Comparison of (**t**) body (n = 8,8,6,7), (**u**) dcLN (n = 11,11,7,8), and (**v**) scLN (n = 15,15,11,12) in i.c.m. injected 5xFAD and WT mice. Data shown are representative of at least two independent experiments using littermate mice. Datapoints in graphs represent individual mice. dcLN weights (**j, n, r, u**) represent an average of both sides (left and right, max one LN per side per mouse) and scLN weights (**k, o, v**) represent an average of all LNs on the left side of the body. *P* values were calculated with (**e, f, j-l, n-p, s, u, v**) two-way ANOVA, (**r, t**) three-way repeated measures ANOVA, and (**i, m**) three-way repeated measures mixed-effects model with Tukey’s post hoc test for multiple comparison. Data are presented as mean values ± s.e.m. Scale bars: 100 μm (**g, h**) and 200 μm (**c, d**).

**Fig. 6:**
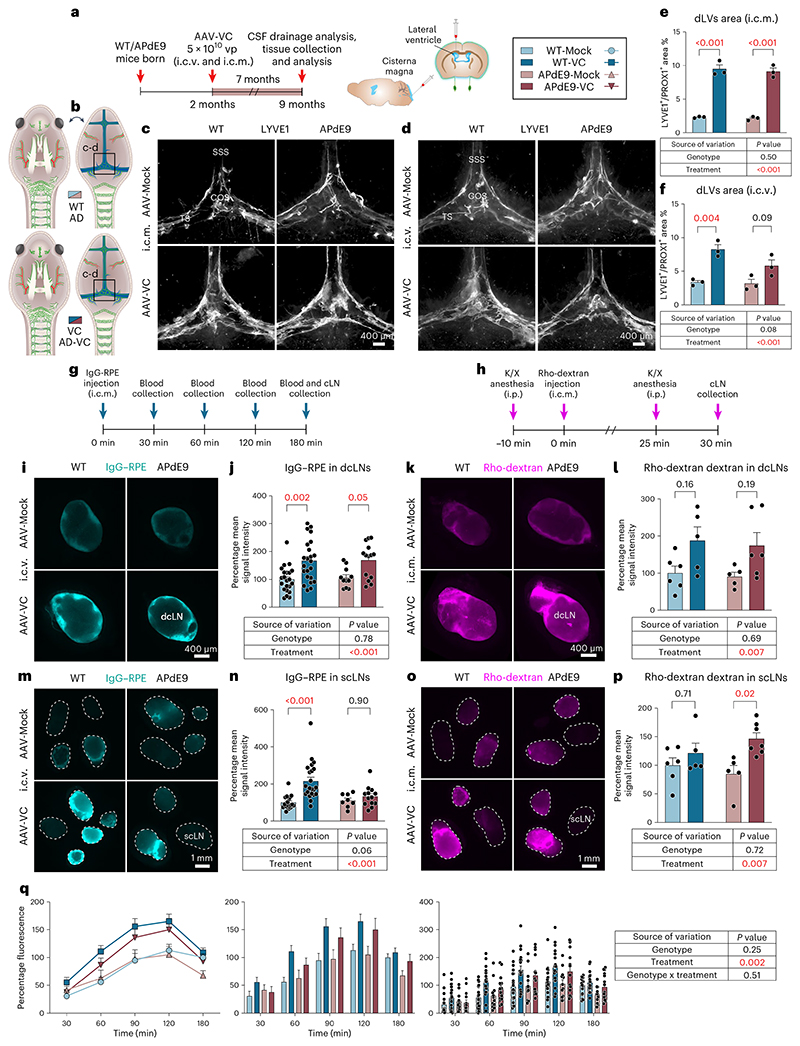
AAV-VEGF-C induced dLV expansion in APdE9 mice improves CSF outflow into cLNs and into blood circulation. Comparison of littermate AAV-Mock and AAV-VC-treated i.c.m. (male) and i.c.v. (female) injected WT and APdE9 mice at 9 months of age. Rho, rhodamin; vp, viral particle; VC, vascular endothelial growth factor C (VEGF-C). **a**, Schedule indicating AAV administration and experimental analysis time points. **b**, Simplified schematic illustration of dLVs (green) attached to the basal and dorsal cranium and spinal canal after removal of the brain and spinal cord. **c**–**f**, Comparison of LYVE1 (white) staining in dorsal skull of i.c.m. (*n* = 4, 3, 3 and 3) (**c,e**) and i.c.v. (*n* = 3, 3, 3 and 3) (**d,f**) injected mice. Pineal gland was excised in (**c,d**) to visualize all dLVs. **g**–**h**, Experimental schedules for IgG–RPE and Rho-dextran drainage analysis into blood (**g**) and lymphatic system (**g,h**). **i**–**l**, Comparison of tracer signal in dcLNs at 180 min (*n* = 22, 23, 11 and 14) (**i,j**) and 30 min (*n* = 6, 5, 5 and 6) (**k,l**) after i.c.m. injection. **m**–**p**, Comparison of tracer signal in scLNs at 180 min (*n* = 20,22,8,13) (**m,n**) and 30 min (*n* = 6, 5, 5 and 7) (**o,p**) after i.c.m injection. **q**, Kinetic analysis of IgG–RPE tracer in systemic blood (saphenous vein) at 30, 60, 90, 120 and 180 min after i.c.m. injection (*n* = 21, 19, 12 and 15), visualized in three different ways. Data shown are representative of at least two independent experiments using littermate mice. Data points shown in graphs represent individual mice. Maximum one LN per side per mouse was used in quantification. IgG-RPE LN values represent an average of both sides. LN and blood tracer signal values were normalized to the average of WT-Ctrl group of every experimental set at 3 h (IgG–RPE) and 30 min (Rho-dextran) time point. *P* values were calculated using two-way ANOVA (**e,f,j,l,n,p**) and three-way repeated measures mixed-effects model with Tukey’s post hoc test for multiple comparison (**q**). Data are presented as mean ± s.e.m. Scale bars, 400 μm (**c**–**d,i,k**) and 1 mm (**m,o**).

To study how the expansion of dLVs by VEGF-C affects AD pathology, we injected 2-month-old WT and APdE9 mice i.c.v. and i.c.m. with 5 × 10^10^ viral particles of AAV-VC or AAV-Mock per mouse and analyzed the mice at 9 months of age (Fig. 6a). Although the AAV-VC-treated mice had a robust dorsal and basal dLV expansion compared to Mock-treated mice (*P* < 0.001; two-way ANOVA), there was again no difference in dLV pattern or area coverage between the WT and APdE9 mice (Fig. 6b–f and Extended Data Fig. 7a–h). Body weights and weights of dcLNs, scLNs or spleen, did not differ between the genotypes or treatment groups (Extended Data Fig. 7i–p). The functionality of the dLVs in the 9-month-old mice was evaluated by studying the accumulation of rhodamine dextran and IgG–RPE to the cLNs at 30 min and 3 h, respectively, after their injection i.c.m. (Fig. 6g,h). The results confirmed that tracer drainage into dcLNs and scLNs was significantly improved by the AAV-VC treatment (*P* = 0.007 at 30 min and *P* < 0.001 at 180 min, two-way ANOVA, about 50–130% increase in predicted mean value), whereas the APdE9 genotype had no significant effect on the drainage into cLNs (Fig. 6i–p). The overall mean IgG–RPE signal intensity in blood was significantly increased in the AAV-VC versus Mock-treated mice (*P* = 0.002; three-way repeated measures mixed-effects model). The overall signal declined in all groups of mice after 120 min (Fig. 6q). Again, the APdE9 genotype did not significantly affect the drainage into blood (Fig. 6q). These results demonstrate that expansion of dLVs by AAV-VC improves CSF macromolecular drainage into both lymphatic and blood vessels, whereas the APdE9 genotype has no effect at 9 months of age.

**Fig. 7:**
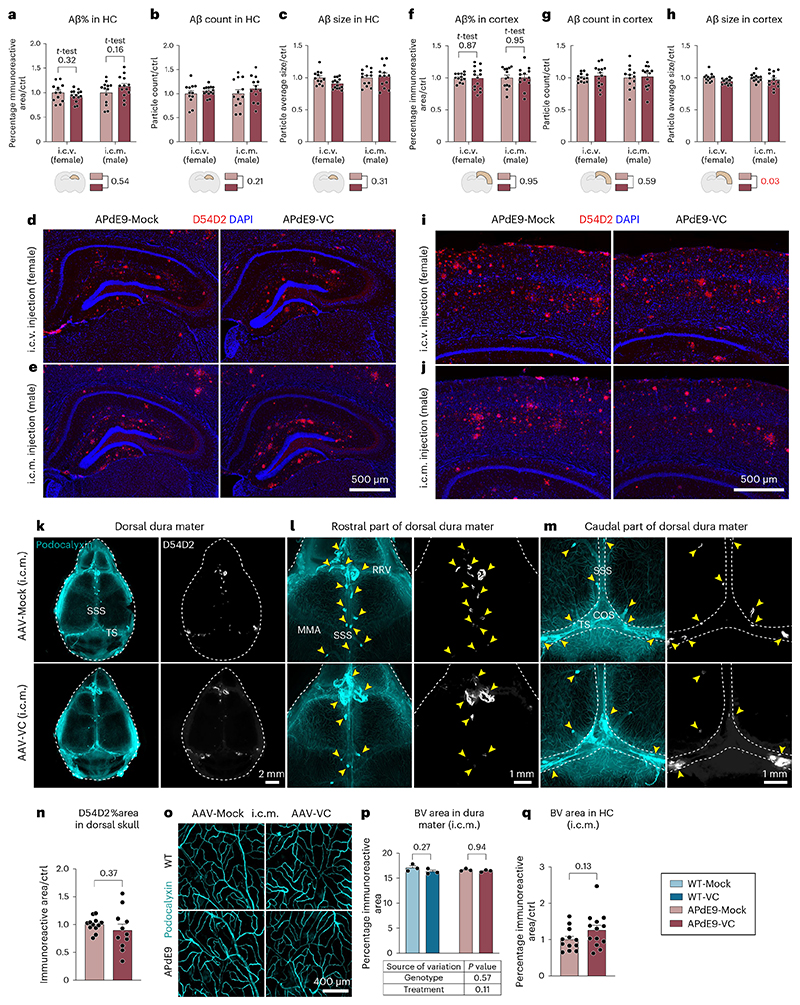
AAV-VC induced dLV expansion does not affect Aβ deposits in brain or dura mater in APdE9 mice. Comparison of littermate AAV-Mock and AAV-VC-treated i.c.m. (male) and i.c.v. (female) injected WT and APdE9 mice at 9 months of age. **a**–**j**, Combined analysis of D54D2 staining (red), including Aβ %, Aβ particle count and Aβ particle average size quantification of i.c.v.- and i.c.m.-injected mice in (**a**–**e**) HC (*n* = 12, 14, 13 and 13) and (**f**–**j**) cortex (*n* = 13, 14, 13 and 13). **k**–**m**, Representative images of podocalyxin (cyan) and D54D2 (white) staining in dorsal skull areas. Yellow arrowheads point to areas where D54D2^+^ Aβ deposits colocalize with podocalyxin^+^ veins connecting to large dural sinuses. Pineal gland was excised in (**k,m**) to visualize all blood vessels. **n**, Quantification of D54D2 area % of dorsal dura mater visualized in **k** (*n* = 12 and 11 combined from *n* = 6 and 5 for i.c.v. and *n* = 6 and 6 for i.c.m.). **o**–**q**, Comparison of podocalyxin (cyan) staining in dorsal dura mater (*n* = 3, 3, 3 and 3) (**o,p**) and in HC (*n* = 12 and 13) (**q**). Data shown are representative of at least two independent experiments using littermate mice. Data points shown in graphs represent individual mice. Brain Aβ and podocalyxin values represent an average of five brain sections (210 mm apart) per mouse. Brain Aβ and podocalyxin as well as dura mater Aβ values are normalized to the average of APdE9-Ctrl group in every experimental set. *P* values were calculated using unpaired two-tailed *t*-test (**a,f,n,q**) and two-way ANOVA with Tukey’s post hoc test for multiple comparisons (**a**–**c,f**–**h,p**). Data are presented as mean ± s.e.m. Scale bars, 400 μm (**n**), 500 μm (**d,e,i,j**), 1 mm (**l,m**) and 2 mm (**k**).

Statistical analysis of the i.c.v.- or i.c.m.-injected APdE9 mice showed no significant differences in the Aβ load in the hippocampus or cortex between AAV-Mock and AAV-VC-treated mice (Fig. 7a-j). The bright anti-Aβ staining in the dura mater was mainly associated with the bridging veins, but not with dLVs (Fig. 7k–m) and was not affected by the AAV-VC treatment (Fig. 7n). Also, AAV-VC treatment did not affect the overall patterning or area of hippocampal or dural BVs (Fig. 7o–q). These data indicate that the AAV-VC-induced sustained increase in dLV function does not affect Aβ accumulation in the brain or dura mater in APdE9 mice.

### dLV expansion by VEGF-C does not alter Aβ load in 5xFAD mice

**Extended Data Fig. 8:**
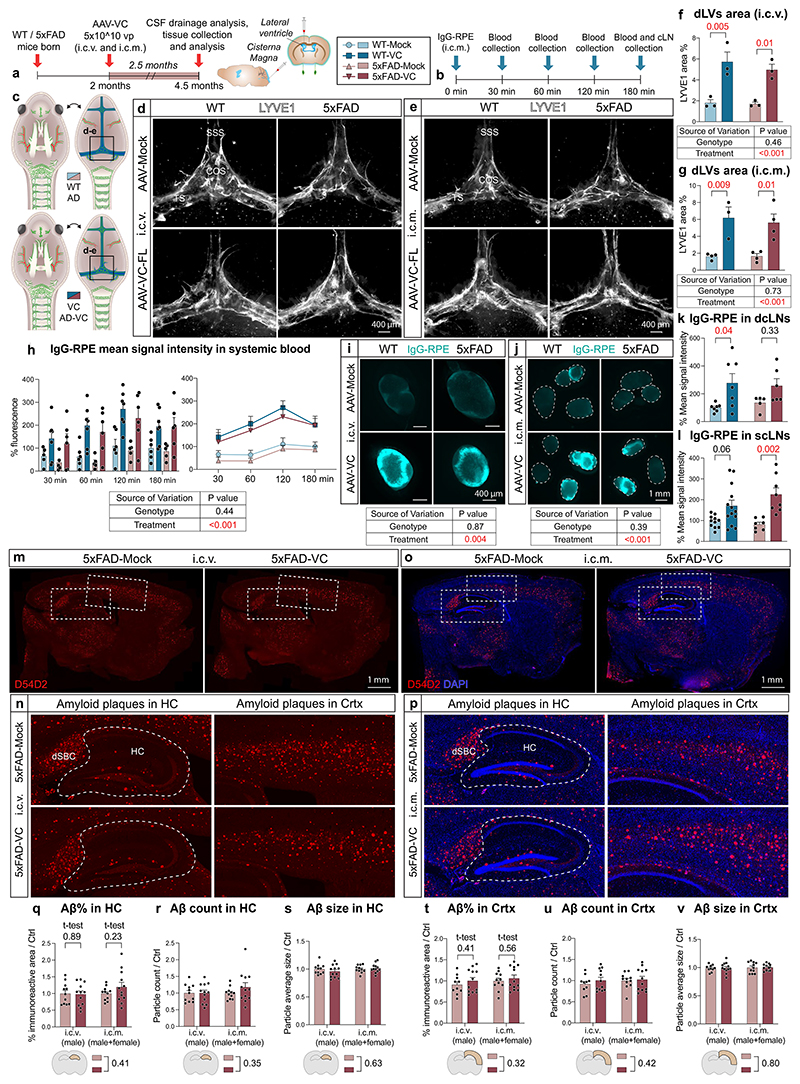
AAV-VC induced dLV expansion in 5xFAD mice improves CSF outflow into cLNs and blood circulation but does not affect Aβ load in the brain. **a-v**, Comparison of littermate AAV-Mock and AAV-VC-treated i.c.m. (male and female) and i.c.v. (male) injected WT and 5xFAD mice at 4.5 months of age. **a**, Schedule indicating AAV administration and experimental analysis time points. **b**, Schedule of CSF drainage analysis. **c**, Simplified schematic illustration of dural LVs (green). **d-g**, Comparison of LYVE1 (white) area percentage in dorsal dura mater after (**d, f**) AAV i.c.v (n = 3,3,3,3) or (**e, g**) AAV i.c.m administration (n = 4,3,4,4). The pineal gland was excised in (**d, e**) to visualize all dLVs. **h**, Kinetic analysis of IgG-RPE tracer in systemic blood at 30, 60, 120 and 180 min after IgG-RPE administration (n = 6,7,5,6) in the i.c.v injected mice visualized by two different ways. **i-l**, IgG-RPE tracer signal in (**i, k**) dcLN (n = 7,7,5,6) and (**j, l**) scLNs (n = 11,13,7,8) 180 minutes after IgG-RPE administration. **m-p**, Comparison of D54D2 (red) staining in hippocampus and cortex after (**m, n**) i.c.v administration and (**o, p**) i.c.m administration. The outlining indicates the quantified HC area without dorsal subiculum (dSBC). **q-v**, D54D2 immunostained area%, particle count and average particle size of the AAV-injected AD mice in (**q-s**) hippocampus (i.c.v. n = 11,12; i.c.m. n = 11,12 from which male n = 6,7; female n = 5,5) and (**t-v**) cortex (i.c.v. n = 10,12; i.c.m. n = 11,12 from which male n = 6,7; female n = 5,5). Data shown are representative of at least two independent experiments using littermate mice. The datapoints shown in graphs represent individual mice. Maximum one LN per side per mouse was used in quantification and dcLN values represent an average of both sides. The IgG-RPE tracer signal in LNs and blood was normalized to the average in the WT-Ctrl group of every experimental set at the 3 h timepoint. Aβ values represent an average of 6 brain sections (400 μm apart) normalized to average of 5xFAD-Ctrl group in every experimental set. *P* values were calculated with (**q, t**) unpaired two-tailed *t*-test, (**f, g, k, l, q-v**) two-way ANOVA and (**h**) three-way repeated measures Mixed-effects model with Tukey’s post hoc test for multiple comparisons. Data are presented as mean values ± s.e.m. Scale bars: 400 μm (**d, e, i**) and 1 mm (**j, m, o**).

To confirm the results in another AD model, 5 × 10^10^ particles of AAV-VC or AAV-Mock were injected i.c.v. or i.c.m. into 5xFAD mice at 2 months of age and the mice were analyzed at 4.5 months of age (Extended Data Fig. 8a). Robust lymphangiogenesis was obtained in AAV-VC-treated mice after both i.c.v and i.c.m. injection versus AAV-Mock-injected mice (*P* < 0.001 in both; two-way ANOVA; Extended Data Fig. 8c–g). Furthermore, AAV-VC significantly improved IgG–RPE drainage from the CSF into dcLNs (*P* = 0.004; two-way ANOVA), scLNs (*P* < 0.001; two-way ANOVA) and to blood circulation (*P* < 0.001; three-way repeated measures mixed-effects model; Extended Data Fig. 8b,h–l). The drainage into blood or cLNs did not differ significantly between 5xFAD and WT mice (Extended Data Fig. 8h–l), neither did the amyloid immunoreactive area percentage in the hippocampus or cortex differ between AAV-Mock- and AAV-VC-injected 5xFAD mice (Extended Data Fig. 8m–v). Furthermore, body or cLN weight was not altered by the 5xFAD genotype or AAV-VC treatment (Extended Data Fig. 7q–v). These data demonstrate that the 5xFAD genotype at 4.5 months does not affect CSF macromolecular drainage into the lymphatic or blood vascular system and that the AAV-VC-induced sustained increase of dLV function does not alter overall brain Aβ load in 5xFAD mice.

### Aβ load in aged AD mice is not affected by dLV expansion

**Extended Data Fig. 9:**
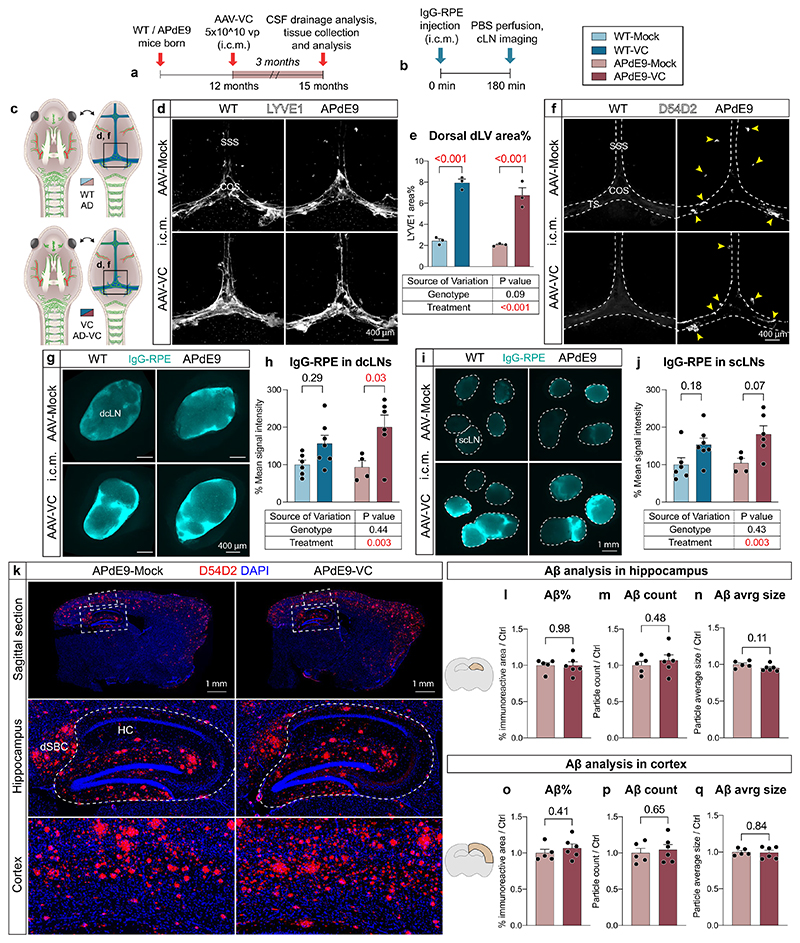
AAV-VC induced dLV expansion in old APdE9 mice improves CSF outflow into cLNs and blood circulation but does not affect Aβ load in the brain. **a-q**, Comparison of data from littermate male WT, APdE9 and AAV-VC-treated (i.c.m.) WT and APdE9 mice at 15 months of age. **a**, Schedule indicating AAV administration and experimental analysis time points. **b**, Schedule of the CSF drainage analysis. **c**, Simplified schematic illustration of dLVs (green) attached to the basal cranium and spinal canal after removal of the brain and spinal cord. **d-f**, Comparison of (**d-e**) LYVE1 (white) and (**f**) D54D2 (white) area in dorsal dura mater (n = 3,3,3,3). The pineal gland was excised from (**d, f**) to visualize all dLVs. **g-j**, Comparison of IgG-RPE tracer signal in (**g-h**) dcLN (n = 7,7,4,6) and (**i-j**) scLNs (n = 7,7,4,6) 180 minutes after i.c.m injection. **k-q**, Comparison of D54D2 staining in (**k, l–n**) hippocampus and (**k, o-q**) cortex of i.c.m injected APdE9 mice (n = 5,6). An example of the quantified HC area without dorsal subiculum is outlined in the images. Data shown are representative of a single experiment using littermate mice. The datapoints shown in the graphs represent individual mice. LN values in (**h, j**) represent an average of both sides (left and right) and maximum one LN per side per mouse was used in quantification. LN IgG-RPE tracer signal values were normalized to the average of WT-Ctrl group of every experimental set at the 3 h timepoint. Aβ values represent an average of 6 brain sections (400 μm apart) per mouse and are normalized to the average of APdE9-Ctrl group of every experimental set. *P* values were calculated with (**l-q**) unpaired two-tailed *t*-test and (**e, h, j**) two-way ANOVA with Tukey’s post hoc test for multiple comparison. Data are presented as mean values ± s.e.m. Scale bars: 400 μm (**d, f, g**) and 1 mm (**i, k**).

**Extended Data Fig. 10:**
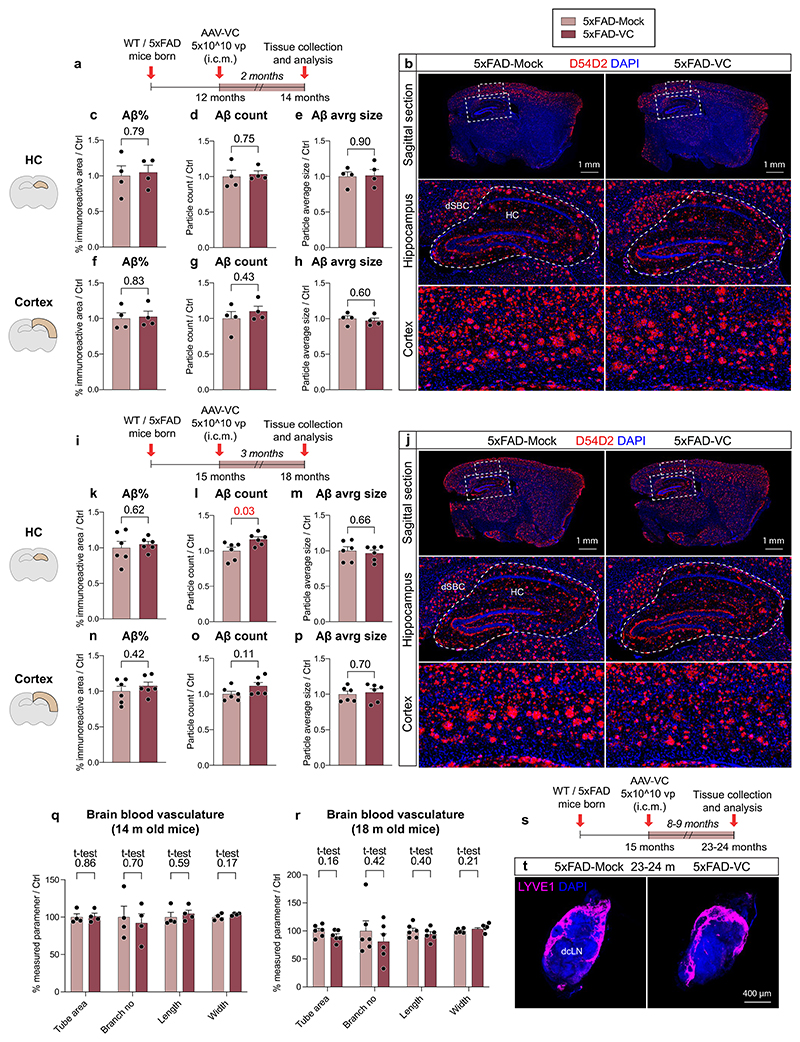
AAV-VC induced dLV expansion in old 5xFAD mice does not affect Aβ load in the brain. **a–t**, Comparison of data from littermate AAV-Mock and AAV-VC treated (i.c.m.) 5xFAD mice at 14 (both sexes), 18 (males) and 23-24 (females) months of age. **a**, Experimental schedule for (**b-h, q**). **a-h**, Comparison of D54D2 (red) staining in (**b–e**) hippocampus and (**b, f-h**) cortex of 14-month-old 5xFAD mice after 2-month AAV treatment (n = 4,4). **i**, Experimental schedule for (**j-p, r**). **j-p**, Comparison of D54D2 (red) staining in (**j-m**) hippocampus and (**j, n-p**) cortex of 18-month-old 5xFAD mice after 3-month AAV treatment (n = 6,6). **q-r**, Quantification of podocalyxin immunoreactive tube area%, branch number, skeleton length, and tube width in cortex of (**q**) 14-month-old 5xFAD mice after 2-month AAV treatment (n = 4,4) and (**r**) 18-month-old 5xFAD mice after 3-month AAV treatment (n = 6,6). Quantification was done with AutoTube software (Montoya-Zegarra et al). **s**, Experimental schedule for (**t**). **t**, Representative image of LYVE1 whole-mount staining in dcLNs of 23-24-month-old 5xFAD mice after 8-9-month AAV treatment (n = 3,3). Data shown are representative of single experiments using littermate mice. The datapoints shown in graphs represent individual mice. Examples of quantified HC areas without dorsal subiculum are outlined in the images (**b, j**). Aβ and podocalyxin values represent an average of 6 brain sections (400 μm apart) per mouse and are normalized to average of 5xFAD-Ctrl group of every experimental set. *P* values were calculated with (**c-h, k-r**) unpaired two-tailed *t*-test. Data are presented as mean values ± s.e.m. Scale bars: 400 μm (**t**) and 1 mm (**b, j**).

To test whether AAV-VC affects already established amyloid plaques, we injected 12-month-old APdE9 mice i.c.m. as above and analyzed the mice at 15 months of age (Extended Data Fig. 9a,b). Again, AAV-VC promoted dural lymphangiogenesis (*P* < 0.001, two-way ANOVA; Extended Data Fig. 9c–e) and improved CSF drainage into cLNs (*P* = 0.003; two-way ANOVA; Extended Data Fig. 9b,g–j), but had no effect on hippocampal or cortical amyloid area percentage (Extended Data Fig. 9k–q). Furthermore, AAV-VC expression for 2 or 3 months in 12- or 15-month-old 5xFAD mice did not affect amyloid plaque load in the hippocampus or cortex or podocalyxin^+^ BV coverage (Extended Data Fig. 10a–r). LYVE1^+^ lymphatic vasculature in the dcLNs did not seem to be affected either by VC treatment (Extended Data Fig. 10s,t). These data demonstrate that, although AAV-VC induces sustained and functional dLV expansion, it does not affect brain Aβ accumulation at an advanced disease stage in APdE9 or 5xFAD mice.

## Discussion

Here we show that sustained manipulation of the major lymphangiogenic growth factor signaling pathway leads to a selective atrophy or hyperplasia of dLVs, but does not cause significant changes in overall brain Aβ plaque load in the two mouse models of AD. Although the AD mice showed typical memory-associated cognitive deficits, dLV atrophy did not change their behavioral scores. AD genotype itself did not affect dLV morphology or function and dural Aβ deposits were associated with bridging veins, but not LVs. The overall CSF-to-blood drainage was not affected by the AD genotype, but unexpectedly, was improved in mice treated with the VEGF-C/VEGF-D trap.

The functional importance of dLVs has been studied by manipulating them either by physical damage or by genetic modifications^20–22,29^. In addition to the method itself, the timing and duration of the treatment also seem to be crucial^20–22,28,29^. To model dLV impairment, we used trapping of the VEGFR3 ligands VEGF-C and VEGF-D, which leads to sustained regression of dLVs that depend on continuous VEGF-C/VEGF-D–VEGFR3 signaling^20,22,43^. In the K14-sR3 mouse model, the VEGF-C/VEGF-D trap arrested LV development in embryos and resulted in nearly complete aplasia of dLVs, both in the dorsal and basal skull. In adult mice, a single i.p. administration of AAV encoding the VEGF-C/VEGF-D trap resulted in complete regression of dorsal and significant atrophy of basal dLVs. To model dLV improvement, we used intracranial AAV-VC treatment of adult AD and WT mice that induced robust lymphangiogenesis in the same areas that also responded to AAV-sR3 treatment. Unlike in a previous study^31^, in our study, VEGF-C delivery induced dural lymphangiogenesis both in the dorsal and basal aspects of the skull and improved CSF drainage in both young and aged mice. The most robust dLV plasticity was seen at sites in which bridging veins connect to dural sinuses and where tracers have been shown to accumulate after i.c.v. and i.c.m. injections^18,22,28,29,44^, possibly because these areas coincide with the CSF drainage pathways.

The physiological functions of dLVs, their connections to upstream and downstream pathways, and the effects of their manipulation are still not fully understood. Our results here, backed up by previous publications, show that macromolecular CSF drainage to cLNs indeed provides an indicator of dLV functionality that can be significantly modified by both VEGF-C blockage and VEGF-C gene delivery^20,22,28,43^. However, the ISF/CSF outflow is regulated by multiple other pathways, various physiological parameters, and shows both site-specific and individual variation. We have previously reported that a macro-molecular tracer injected into brain ISF is drained via dLVs preferentially into ipsilateral cLNs, while injection into the CSF space leads to outflow into cLNs on both sides of the neck^20^. Individual differences in cLN connections and drainage features downstream of dLVs have been also reported^18^, which could partly explain the individual variation in macromolecular CSF outflow to the cLNs and to blood even though the dLV atrophy and expansion were relatively consistent in our experiments.

Notably, both growth and regression of dLVs resulted in improved macromolecular CSF-to-blood drainage in APdE9 mice and their WT littermates. Based on previous publications and our results here, the improved drainage in AAV-VC-treated mice likely depends on the robust dural lymphangiogenesis that induced increased transfer of the macromolecular tracer, in a sequential manner, first into cLNs and later into blood circulation^31,45^. Consistently with this, the dLV atrophy in the AAV-sR3-treated mice impaired tracer drainage into the cLNs, but notably, increased CSF-to-BV drainage. Because the skull is a rigid structure, the impaired fluid drainage due to dLV atrophy is expected to lead to increased ICP or activate other pathways that can increase CSF efflux. K14-sR3 mice, which have dLV atrophy, show no change in ICP^20^, a finding that we extend here to AAV-sR3-treated mice. These results indicate that CSF fluid outflow dynamics can indeed compensate for the loss of LVs to maintain pressure homeostasis inside the skull. In our search for possible compensatory mechanisms, we showed that mice expressing the VEGF-C/VEGF-D trap did not have (1) brain ventricle enlargement; (2) changes in hippocampal aquaporin-4-positive astrocytic end feet that form a part of the BBB perivascular pathway; or (3) changes in hippocampal BV coverage. A more comprehensive mechanistic exploration of the various ISF/CSF outflow pathways is required for assessment of their relative contributions to CSF outflow and responses to dLV modulation.

The overall brain Aβ accumulation was not significantly affected by lymphangiogenic growth factor manipulation of the dLVs. This suggests that even though dural LVs are capable of draining macromolecules, they are not actively involved in Aβ transport from the brain in the AD mouse models that we used. Moreover, the amyloid deposits in the dura mater were mostly confined to the bridging veins that connect brain to large dural sinuses and were not affected by dLV manipulation, suggesting that these blood vessels and/or their perivascular space, but not dLVs, are primarily involved in the transport of brain-derived Aβ into the dura mater. Furthermore, the AD genotype did not significantly alter the dLV coverage or macromolecular CSF tracer drainage to cLNs, even when advanced brain and dura mater amyloidosis was apparent. Our results do not mean that ISF/CSF efflux as such would not affect the amyloid deposition in AD, but they call for a critical assessment of direct dLV involvement in Aβ drainage. The lack of dLV manipulation effect on brain Aβ accumulation may be partly explained by the high Aβ42/Aβ40 ratio in the two AD models we used. In patients with AD, the shorter and less-aggregation-prone Aβ40 peptide is the dominant product, whereas in the APdE9 mice, the Aβ42/Aβ40 ratio is above 1 (~1.3)^37^ and in the 5xFAD mice, above 5 (up to 25 in young mice^41^). A high proportion of the aggregation-prone Aβ42 leads to rapid deposition of Aβ into the brain parenchyma and formation of compact Aβ plaques that resist clearance. So far, only a few studies have reported Aβ in cLNs in AD mouse models^26^ or in humans^27^. Future studies with animal models that better mimic the human Aβ42/Aβ40 ratio should be used to address the LV function in the CNS-produced Aβ clearance.

In agreement with our findings, a recent report found no significant differences in dLV morphology, area coverage or CSF-to-dcLNs accumulation between young AD mice (2–4-month-old 5xFAD or APP-J20 mice) and their WT controls^31^. Furthermore, recent reports have shown Aβ staining in the dorsal dura mater after verteporfin-mediated photodynamic destruction of dorsal dLVs, but not within dLVs or cLNs^31,32^. The latter paper also reported decreased dLV coverage in some dorsal skull areas of older AD mice (13–14-month-old 5xFAD mice) versus their age-matched WT controls^32^. Even though we and others have previously reported that aging itself is associated with disjointed, sparse and hyperplastic dLVs that show fewer valves than in young mice^22,28,31^, we did not detect significant AD-associated alterations in dLV morphology or CSF drainage into cLNs at the analyzed ages. Furthermore, in contrast to the previous report showing Aβ deposition in the dura mater of 5xFAD (3–4 months) and APP-J20 (7–8 months) mice mainly after verteporfin-mediated photodynamic destruction of dorsal dLVs^31^, we found such Aβ deposits in unmanipulated 5xFAD and APdE9 mice of similar age. These differences could reflect variability in pathogenesis between AD mice or differences in the analysis methods.

Our findings differ from those in the recent reports where verteporfin-mediated photodynamic destruction of dorsal dLVs aggravated Aβ accumulation in the brains of 5xFAD and APP-J20 mice^31,32^. As the AD models we used are either the same, or very similar to those used in these two reports, the major differences between the findings in the brain amyloid pathology relate most likely to the different methods to target the dLVs. Cranial photodynamic ablation employs intracranial injection of photoconvertible verteporfin followed by laser treatment at five spots through the intact dorsal skull with the aim to acutely destroy dorsal cranial dLVs; this is repeated 2–4 times in prolonged experiments^29,31,32^. When injected i.c.m., verteporfin distributes to CSF, ending up also in dLVs on its way to the cLNs. When illuminated with laser light, verteporfin generates reactive oxygen species that result in rapid local damage to and necrosis of the cells in close vicinity of the laser-activated verteporfin^46–51^. The ablation results in partial destruction of dorsal dLVs and decreased macromolecular CSF-to-dcLN clearance^29,31,32^. However, verteporfin-mediated destruction of the lymphatic endothelium has been shown to induce off-target/bystander effects, including the death of smooth muscle cells and upregulation of inflammatory markers^29,46^. The possible inaccuracy of laser targeting through the skull and the bystander and inflammatory effects of verteporfin may also contribute to intracranial Aβ accumulation, especially as inflammation is known to contribute to AD pathogenesis^2^. Unlike verteporfin, a single injection of AAV encoding the VEGF-C/VEGF-D trap leads to apoptosis of lymphatic endothelial cells due to growth factor deprivation and sustained regression of dLVs both in the dorsal and basal skull^22,28^. As discussed above, the more gradual regression of dLVs may allow activation of mechanisms that compensate the decreased fluid outflow. However, the verteporfin-mediated photoablation of dorsal dLVs did not lead to increased ICP, at least when measured 3 d after ablation^29^, suggesting that compensation mechanisms are also active there. Definition of such mechanisms requires improved knowledge on CSF outflow pathways and how these are altered during perturbations of CSF homeostasis such as the abrupt verteporfin ablation and the more gradual growth factor deprivation-induced regression.

Even though we could not affect AD-related amyloid pathology by manipulation of the VEGF-C pathway, it is possible that this in different disease models or when combining VEGF-C with other compounds affecting LVs, such as Piezo1 agonists^52^, or known disease-modifying treatments, could result in translationally significant outcomes. Indeed, VEGF-C treatment has been suggested to improve cognition in older mice^31^, to have synergistic effect with PD-1 treatment to eradicate glioblastoma^45^, to rescue dLV defects in a craniosynostosis mouse model^53^, to alleviate effects of CNS viral infection^54^ or traumatic brain injury-induced gliosis^55^, as well as to aid the recovery from stroke^56^ and intracerebral hemorrhage^57^. Conversely, the effect of the VEGF-C/VEGF-D trap is currently being evaluated in a phase 3 clinical trial on wet-type age-associated macular degeneration and in a phase 2 clinical trial on diabetic macular edema (ClinicalTrials.gov identifier NCT02543229). However, it is important to acknowledge that some of the effects aiming for specific dural LV manipulation might be related to manipulation of extracranial LVs or non-lymphatic endothelial cells. For example, VEGF-C can also activate brain neural stem/progenitor cells^58^, expand and improve the function of nasopharyngeal lymphatic plexus^59^ and increase collecting LV pumping by stimulation of the contractility of smooth muscle cells around the LVs^60^.

Our results suggest that although dLVs function in the macro-molecular CSF-to-cLNs drainage, they do not actively drain Aβ deposits from the brain in the two mouse models with high levels of aggregation-prone Aβ42. Thus, the overall brain Aβ accumulation could not be significantly affected by lymphangiogenic growth factor manipulation of the dLVs. Furthermore, our findings emphasize the fact that the overall CSF drainage is regulated by multiple pathways that can compensate each other and sustain sufficient overall CSF efflux during dLV atrophy. These findings highlight the need for further comparison of different methods to manipulate the dLVs and for better mechanistic understanding of CSF circulation and outflow in physiological and pathological conditions.

## Methods

### Animal study approval

The study was approved by the Animal Experiment Board of Finland.

### Mice

We used the following transgenic mouse lines: K14-VEGFR3_1–3_-Ig (C57BL/6JOlaHsd background ^36^), APdE9 (B6. Cg -Tg(APPswe,PSEN1dE9)85Dbo/Mmjax, from Jackson Laboratories, MMRRC stock no. 34832-JAX^37^), 5xFAD (B6.Cg-Tg(APPSwFlLon,PSEN1*M 146L*L286V)6799Vas/Mmjax, from Jackson Laboratories, MMRRC stock no. 34848-JAX^41^), Rosa26^LSL-TdTomato^ (C57BL/6JRj background^61^, from Jackson Laboratory, stock no. 021875), BmxCreER^T2^ (C57BL/6JRj background^62^) and littermate control mice and WT mice on a C57BL/6J (000664) or C57BL/6JOlaHsd background. Supplementary Table 2 lists the genotyping oligonucleotide sequences. Because of the nature of disease progression in different AD models, AAV treatment, behavioral analysis and experimental ending were assessed within a ± 6-d age range in the APdE9 model and within a ± 2.5-d age range in the 5xFAD model. Cre-recombinase was activated by two consecutive daily doses of tamoxifen (2 mg, 579002, Sigma-Aldrich) in corn oil.

For behavioral experiments, the mice were transferred into individually ventilated cages (Mouse IVC Green Line; air change 75 times per h with maximal airspeed 0.05 m s^−1^; half of the cage covered by a wire bar food hopper; Tecniplast). Cage enrichment was provided by bedding (aspen chips, 4HP), nesting material (aspen strips, PM90L) and an aspen brick, all from Tapvei. Food pellets (Global Diet 2916C, Envigo) and water were available ad libitum. Room temperature was 22 ± 2 °C and relative humidity was 50 ± 15%. The lights were on from 6:00 to 18:00, when the experiments were carried out.

### AAV transduction

The AAVs of serotype 9 were produced by the AAV gene transfer and cell therapy core facility^63,64^.

For i.p. AAV transduction, adult C57BL/6J mice (8–10 weeks old) received a single i.p. dose (1 × 10^12^ vp in 200 μl) AAV encoding the ligand-binding domains 1–4 of VEGFR3, fused to the IgG Fc domain (AAV-mVEGFR3_1–4_-Ig)^65^. Control mice received the same dose of AAV encoding the domains 4–7 of VEGFR3 fused to the IgG Fc domain (AAV-mVEGFR3_4–7_-Ig)^66^.

For intracranial AAV transduction, a single dose (1 × 10^10^, 5 × 10^10^ or 10 × 10^10^ viral particles (vp) per mouse in 4 μl) of AAVs encoding VEGF-C^67^ or without payload was injected i.c.v. or i.c.m. according to previously published methods^20,22^. The i.c.v. was performed unilaterally (coordinates, A/P −0.34; M/L 1.0; D/V −2.5 mm below dural surface) with the aid of the mouse brain atlas of Paxinos and Franklin^68^. After proper exposure of the injection site, the needle was slowly lowered into the correct position (lateral ventricle or i.c.m.) and kept in place for 2 min before 4 μl per mouse was injected using a 33-G, 10-μl, microsyringe (NanoFil; World Precision Instruments) coupled to a stereotactic microinjector (Stoelting) at 0.5 μl min^−1^. The needle was kept in place for an additional 4 min to avoid a leakage, then slowly retracted and skin was closed with metallic clips. After subcutaneous carprofen (5.0 mg kg^−1^) injection, mice were transferred to a warm chamber until they were fully awake.

### CSF drainage analysis

Mice were injected intracranially with IgG–RPE (1 μg μl^−1^ in 4 μl, Thermo Fisher, PA1-86078) or Rho-dextran (6 μg μl^−1^ in 4 μl, 70 kDa) and placed into a warm chamber on the same side.

For CSF-to-blood analysis, blood was collected from the saphenous vein at the indicated time points. At the experimental end-point (180 min), blood was collected from the heart and mice were perfused with phosphate-buffered saline (PBS) as indicated below. For IgG–RPE fluorescence detection, 5 μl serum was diluted with 95 μl PBS and detected at 560/580 nm (ex/em) (EnSight Multimode Plate Reader, PerkinElmer). The final values represent an average of two runs after subtraction of background signal from PBS. Mice with signs of unsuccessful injection (tracer visible in cerebellum after i.c.m. injection) and unsuccessfully collected blood samples (values below detection level) were omitted from analysis.

For CSF-to-LN analysis, the mice were PBS perfused and the LNs were collected at 30 min (Rho-dextran) or 180 min (IgG–RPE) after injection, immersed in ice-cold 4% PFA, fixed overnight at +4 °C, washed in PBS and imaged without delay. Before imaging, the LNs were carefully dissected from the fat and placed on same side under the light microscope. Fluorescent stereo micrographs of tracer within LNs were obtained by using the AxioZoom V16 fluorescence stereo zoom microscope (Carl Zeiss) with an OptiMOS sCMOS camera (QImaging) and ZEN 2012 software (Carl Zeiss) for image acquisition. Imaging was conducted using the same exposure and magnification settings for the same set of LNs. The LNs with brightest fluorescence signal were always chosen for analysis so that maximum one LN per side (left and right) per mouse were included and final values represent an average of both sides. In ImageJ software (National Institutes of Health), the chosen LNs were outlined manually and mean tracer signal intensity within the LN area was quantified for every chosen LN. If multiple different experimental sets were included in analysis, they were pooled together by normalizing to the average of a suitable control group in each experimental set.

### Magnetic resonance imaging

Anatomical MRI images were acquired in a 7T/16-cm horizontal Bruker Pharmascan system with a standard Bruker quadrature resonator volume coil and a mouse brain quadrature surface coil. A three-dimensional (3D) multi gradient echo sequence was used with the following parameters: TR = 68 ms, TE = 2.73 ms, echo spacing 2.9 ms, echoes 13, flip angle 16° and matrix size 125 μm^3^. The anatomical images were intensity bias-corrected using N4BiasCorrection from Advanced Normalization Tools (http://stnava.github.io/ANTs/)^69^. Ventricle masks were created by intensity thresholding and the number of voxels and the volumes in mm^3^ were computed in each animal.

### ICP measurements

Animals were anesthetized using ketamine (100 mg ml^−1^) and xylazine (10 mg ml^−1^), their heads fixed on a stereotaxic frame and a 30-G dental needle connected to a PE10 tube filled with saline was used to puncture the cisterna magna. The tube was connected to a pressure transducer (BP-102, iWORX) coupled with an iWORX data acquisition system (iWORX IX-RA-834) and Labscribe 4 software. ICP was measured for 5 min and average value was reported.

### Tissue collection

After a lethal dose of ketamine and xylazine, the mice were perfused through the left ventricle with ice-cold PBS for 3–4 min at 8 ml min^−1^ after puncture of the right auricle. Head dissection and brain and skull fixation were conducted as previously published^22^. For general IHC, the brain and LNs were cryoprotected with 30% sucrose in PBS at least overnight, frozen with liquid nitrogen and/or dry ice and stored at −80 °C before cutting. For IHC analysis of BVs, the brain hemispheres were frozen by immersion into a container filled with 2% isopentane in 2-methylbutane placed in dry ice. For protein/RNA analysis, the dissected tissues were cut into suitable blocks, snap frozen in liquid nitrogen and stored at −80 °C before analysis.

### Whole-mount immunostaining

Whole-mount immunostaining of the dura mater was carried out as previously described^22^. For confocal microscopy, the skullcaps were decalcified after whole-mount staining with EDTA for 1–2 d, mounted in Vectashield fluorescent medium (Dako) or Prolong Gold antifade reagent (Invitrogen) between a glass slide and a cover glass, then sealed with Cytoseal 60. To gain best visualization of BVs, LVs and Aβ deposits, dorsal dura mater samples were first whole-mount stained with primary antibodies against goat/rat podocalyxin (BVs) and rabbit anti-Aβ 1-37/42 (Aβ deposits, clone D54D2) and detected with Alexa Fluor 488 and 647 secondary antibodies, respectively. Next, the samples were stained with rabbit anti-mouse LYVE1 (LVs) and detected with Alexa 594 secondary antibody. Because Aβ deposits were occasionally visible in the LYVE1 channel, separate staining with D54D2 and LYVE1 and co-staining with D54D2 and PROX1 was used to confirm that D54D2 was not, in general, associated with LYVE1^+^/PROX1^+^ dural LVs. The bridging veins connecting brain and dura were torn when dorsal skullcap was detached from the brain for whole-mount staining.

For immunofluorescent (IF) whole-mount staining LNs, a modified version of the SUMIC protocol was applied. After perfusion, freshly dissected LNs were rinsed with PBS and fixed for 3 h in ice-cold 4% PFA and 0.05% glutaraldehyde. After washing with PBS, tissues were dehydrated in an ice-cold methanol gradient of 50%, 80%, 100% and 100% for 40 min each. Next, LNs were bleached by immersion in 5% (*v*/*v*) H_2_O_2_ in methanol for 3 h and rehydrated in a reverse methanol gradient. After washing with PBS, antigen retrieval and permeabilization were performed by incubating LNs at 4 °C for 12 h in ice-cold buffer containing 25% urea, 15% glycerol and 7% Triton X-100 (TX) diluted with ddH_2_0. Next, LNs were digested in freshly prepared 0.2% Collagenase A in PBS for 30 min at 37 °C on a shaker. After washing with 2% FBS in PBS, the LNs were transferred into tubes and blocked for 20 min at 37 °C in a freshly prepared mix of 10% donkey serum, 10% dimethylsulfoxide (DMSO) and 0.5% TX in PBS. Next LNs were incubated overnight (o/n) in primary antibody solution in 2% *v*/*v* donkey serum, 10% DMSO and 0.5% TX in PBS at 37 °C in a rotating chamber. After 3 h washing with 2% *v*/*v* donkey serum and 0.5% TX in PBS at 37 °C, LNs were incubated with Alexa Fluor-coupled secondary antibodies in 2% *v*/*v* donkey serum, 10% DMSO and 0.5% TX in PBS for 8 h at 37 °C. After 3 h washing with 2% *v*/*v* donkey serum and 0.5% TX in PBS at 37 °C, LNs were dehydrated in an 30%, 50%, 80% and 100 % gradient of isopropanol for 30 min each, rinsed twice with ethyl cinnamate (Sigma-Aldrich, cat. no. 112372) for 5 min each and cleared with 80% ethyl cinnamate and 20% PEGM (Sigma, cat. no. 447943) under gentle rotation at room temperature for 30 min. After clearing, samples were placed inside Vecta-shield-filled silicon wells attached to a glass slide, sealed with Cytoseal 60 and imaged as soon as possible. Please refer to Supplementary Table 1 for antibodies used.

### Immunostaining of cryosections

For IF staining of cryosections, 10-μm (LNs), 20-μm (sagittal brain), 35-μm (coronal brain) or 50-μm (coronal brain) thick sections were cut using a Cryostar NX70 (Thermo Scientific). Brain hemispheres frozen in 2% isopentane in 2-methylbutane and all LNs were cut directly onto a glass slide and stored at −20 °C before staining. The cryoprotected brain sections were stored as free-floating sections in a 24-well plate filled with antifreeze solution, stored at −20 °C and, before staining, lifted onto slides and washed with PBS at least four times.

For IF-based LN and brain staining, cryosections were air-dried, fixed with 1–4% PFA for 5 min (only brain hemispheres frozen with isopentane), washed in PBS and permeabilized with 0.3% PBS–TX. Brain sections for IF for anti-Aβ W02 staining underwent antigen retrieval before permeabilization by incubation in 10 mM citrate buffer for 20–30 min at 85 °C followed by washes with PBS. After blocking with DIM, the samples were incubated overnight at 4 °C with primary antibodies in DIM. After washing with 0.1% PBS–TX, the sections were incubated with fluorescent dye-conjugated secondary antibodies diluted at 1:500 in DIM for 1 h at room temperature. After washing with 0.1% PBS–TX and PBS, the sections were post-fixed with 1% PFA and mounted in Vectashield hard set fluorescent mounting medium (Dako) or Prolong Gold antifade reagent (Invitrogen) between a glass slide and cover glass.

For DAB-based free-floating staining of thick brain cryosections, sections were cut and stored as above, lifted on slides, air-dried, washed with PBS, incubated in 10 mM citrate buffer for 20–30 min at 85 °C for antigen retrieval, followed by washing with 0.5% TX in Tris-buffered saline (TBS; pH 8.6) and primary antibody incubation in TBS–TX on a shaker in the dark, overnight at room temperature. After washing with TBS (pH 8.6), the sections were incubated with biotin-conjugated secondary antibodies diluted at 1:500 in TBS–TX for 2 h at room temperature, followed by washing with TBS–TX and incubation with Streptavidin diluted 1:1,000 in TBS–TX for 2 h. After washing with TBS–TX, the sections were incubated in metal-intensified DAB (20 ml 0.05 M Tris buffer, pH 7.6, 5 mg DAB, 1 ml saturated Ni ammonium sulfate solution and 15 μl 40% H_2_O_2_) for 3 min, washed with PBS and mounted with Vectashield hard set (Dako) or Prolong Gold antifade reagent (Invitrogen). Please refer to Supplementary Table 1 for antibodies used.

### Microscopic image acquisition, processing and analysis

IHC staining was analyzed by a researcher that was blinded to the genotype and treatment of the mice.

Fluorescent stereo micrographs of stained skull whole-mount samples were obtained by using the AxioZoom V16 fluorescence stereo zoom microscope with the ZEN 2012 software for image acquisition. For dura mater samples, LYVE1^+^/PROX1^+^ or LYVE1^+^/Podoplanin^+^ LVs, Podocalyxin^+^ BVs and D54D2^+^ amyloid areas were highlighted manually and quantified using the thresholding tool of the ImageJ software. Highlighted dural LVs, BVs and amyloid areas were reported as area coverage of the region of interest. If multiple different experimental sets were included in analysis, they were pooled together by normalizing to the average of a suitable control group in each experimental set.

Laser scanning confocal micrographs of the fluorescently labeled LN wholemounts, LN cryosections and skullcap flat mounts were obtained using an LSM 780 confocal microscope (Carl Zeiss) with multichannel scanning in frame (air objectives ×10 Plan-Apochromat with NA 0.45 and ×20 Plan-Apochromat with NA 0.80). Zen 2011 software (Carl Zeiss) was used for image acquisition. The resulting micrographs were rendered to maximum intensity projections.

Micrographs of DAB-based anti-Aβ W02-stained brain sections were imaged with a NanoZoomer XR slide scanner (Hamamatsu Photonics) at ×40 (corresponding to 0.23 μm per pixel), viewed with NDP.view 2 software (Hamamatsu Photonics) and exported in .TIF format into ImageJ software for quantitative analysis. For anti-Aβ W02 staining, a series of adjacent coronal sections of one hemibrain (35-μm sections, five sections per mouse, 210 μm apart from each other) were stained. Two regions of interest were hand-drawn, the hippocampus excluding subiculum and the overlying cerebral cortex from the midline (90°) to the widest point of the section (0°). The threshold for the signal detection was first determined by a random sample set so that all identified amyloid plaques were detected with the minimum number of artifacts and noise in the background. Once defined, the same threshold was used in all sections. Highlighted Aβ areas were measured and reported as area fraction of the region of interest. A minimum size of 20 pixels was applied for the amyloid plaque analysis.

Fluorescent micrographs of anti-Aβ D54D2, BV podocalyxin and aquaporin-4 (AQP4)-stained brain sections were obtained by using a Pannoramic 250 Flash III fluorescence slide scanner (3DHISTECH) with ×20 (NA 0.8) air in FIMM Digital Microscopy and Molecular Pathology Unit. Images were viewed with CaseViewer 2.4 software (3DHISTECH) and exported in .TIF format into ImageJ software for quantitative analysis. For D54D2 and podocalyxin staining of AAV-treated animals, a series of adjacent sagittal sections (20-μm sections, six sections per mouse, 400 μm apart from each other) or coronal sections (35-μm sections, six sections per mouse, 210 μm apart from each other) of one hemibrain were double stained for D54D2 and podocalyxin. For hippocampal astrocytic end feet analysis, a series of adjacent coronal sections (35-μm sections, five sections per mouse, 210 μm apart from each other) were stained for anti-aquaporin-4 (AQP4). For hippocampal BV staining of K14-sR3 versus WT mice, a series of adjacent sagittal sections (50-μm sections, two sections, 200 μm apart from each other) were stained for podocalyxin. The regions of interest (hippocampus and cortex) were drawn as presented above. Using ImageJ, podocalyxin^+^ brain BVs, D54D2^+^ amyloid areas and AQP4^+^ astrocytic end feet were highlighted from the region of interest using the thresholding tool. In the D54D2 and podocalyxin analysis of the AAV-treated mice, the threshold was determined as presented above and the same threshold was used within the same experimental set. In AQP4 analysis, the threshold was applied manually for every sample by the same person in one quantification session. For hippocampal BV quantification in the K14-sR3 versus WT mice, an average value of four different ImageJ software automated threshold tools (namely Li, Moments, Otsu and Triangle) was used for final comparison. Highlighted Aβ, podocalyxin and AQP4 areas were measured and reported as the area fraction of the region of interest. The average size and number of amyloid particles were quantified from the region of interest using particle size 10–infinity (inch^2^) and circularity 0–1, with holes included in the analysis. Podocalyxin immunoreactive tube area, branch number, skeleton length and tube width values in cortex of older AAV-Mock-versus AAV-VC-administered 5XFAD animals were quantified by using AutoTube software^70^.

### Brain Aβ ELISA

Deep-frozen blocks of hippocampus and parieto-occipital cortex of one hemisphere from APdE9;K14-sR3, AAV-sR3 injected APdE9 and their control APdE9 mice were weighed and homogenized in 10× volume of PBS, containing complete inhibitory mixture (Roche Diagnostics). Samples were centrifuged at 181,000*g* for 2 h at 4 °C. The remaining pellet was resuspended in 8× the original volume of 5 M guanidine-HCl/50 mM Tris-HCl, pH 8.0 and mixed on a shaker for 3 h at room temperature. Samples were then diluted 1:25 with reaction buffer (Dulbecco’s PBS with 5% bovine serum albumin and 0.03% Tween-20, supplemented with a protease inhibitor cocktail) and centrifuged at 15,700*g* for 20 min at 4 °C. Decanted supernatant was further diluted 1:500 with dilution buffer and used for analysis of insoluble Aβ40 and Aβ42 species, estimated using ELISA kits (Biosource International) according to the manufacturer’s instructions. Aβ40 and Aβ42 levels were standardized to brain tissue weight and expressed as picograms of Aβ per gram ± s.e.m.

### Western blotting

VEGFR3_1–4_-Ig and VEGFR3_4–7_-Ig proteins in serum were detected by western blotting using antibodies against the extracellular domain of VEGFR3 (1:1,000 dilution; R&D, AF743). Mice without expression (demonstrated in Fig. 1b) were omitted from analysis.

### Quantitative real-time PCR

Cortex samples of PBS-perfused mice were snap frozen in liquid nitrogen. Total RNA was extracted using TRIsure (Bioline) with phenol–chloroform followed by column isolation using a Nucleospin RNA kit (Macherey Nagel). Complementary DNA was synthesized from 500 ng total RNA using the High-Capacity Reverse Transcription kit (Applied Biosystems). Quantitative real-time PCR was performed using the following primer pairs; *Vegfc* F: 5′*-*GAGGTCAAGGCTTTTGAAGGC*-*3′, *Vegfc* R: 5′-CTGTCCTGGTATTGAGGGTGG*-*3′; *Rplp0* F: 5′-GGACCCGA-GAAGACCTCCTT*-*3′, *Rplp0* R: 5′-GCACATCACTCAGAATTTC*-*3′. The qPCR reactions were carried out with FastStart SYBR green master mix (Roche) and a Bio-Rad C1000 thermal cycler, according to a standardized protocol, and gene expression fold changes were calculated using the 2^–ΔΔCT^ method. Please refer to Supplementary Table 2 for sequences of the oligonucleotides used for qPCR.

### Nest construction

After changing the cage to a clean one, the standard nesting material (aspen strips, PM90L, Tapvei) was replaced by two pieces (5-cm square, ~2.5 g) of compressed cotton fiber (Nestlets, Ancare) was added to the cage. The next morning (NestScore1, ~17 h later), the nests were assessed by visual inspection on a rating scale of 1–5 according to a previously published protocol (1, Nestlet >90% intact, no visible piling of bedding material, no shredded cotton; 2, Nestlet >50% intact, slight piling of bedding and/or small crater; 3, Nestlet mostly shredded (<50% intact) but not in identifiable nest site, the cotton is not gathered into a nest but is spread around the cage; 4, identifiable but flat nest, more than 90% of Nestlet is shredded; 5, crater-shaped nest)^71^. Assessment was repeated 24 h later (NestScore2) then the nest was removed and standard aspen strips were returned to the cage. Unused Nestlet was weighed.

### Light–dark exploration

The test was carried out in the square open field arena (30 × 30 × 20 cm, Med Associates) equipped with infrared light sensors detecting horizontal and vertical activity. The dark insert (non-transparent for visible light) was used to divide the arena into two halves. An opening (a door with a width of 5.5 cm and height of 7 cm) in the wall of the insert allowed free movement of mice from one compartment to another. Illumination in the center of the light compartment was ~550 lx. The animal was placed in the dark compartment and allowed to explore the arena for 10 min. Latency to enter the light side, distance traveled, number of rearings and time spent in different compartments were recorded by the program (Activity Monitor, v.5.8, Med Associates). The number of fecal boli was counted at the end of the trial.

### Open field

Four 50 × 50-cm square arenas (made of white PVC) were placed under a camera for tracking animals by Ethovision XT13 (Noldus). Mice were released in one of the corners and monitored for 20 min. Between trials, the arenas were cleaned with water. The distance traveled and time spent in the center (40 × 40-cm area) were used for analysis.

### Morris water maze

The system consisted of a white circular wading pool (Ø 120 cm) and a transparent escape platform (Ø 10 cm) submerged 0.5 cm under the water surface in the center of one of four imaginary quadrants. The animals were released to swim in random positions facing the wall and the time to reach the escape platform (maximum time 60 s) and the swimming distance were measured in every trial with Ethovision XT13 video-tracking (Noldus). In addition, thigmotaxis, the time spent swimming within the outermost ring of the pool (10 cm from the wall) was measured. Two training blocks consisting of three trials each were conducted daily. The interval between trials was approximately 5 min and the time between training blocks was about 5 h. The hidden platform remained in a constant location for 3 d (six initial training sessions; counterbalanced between individuals) and was thereafter moved to the opposite quadrant for the next day (two reverse training sessions). The probe trial was conducted approximately 18 h after the last initial training session (in the morning of the fourth day). In the probe trial, the mice were allowed to swim in the maze for 60 s without the platform being available. Spatial memory in the probe trial was estimated by preference to the trained region (imaginary circular area of Ø 30 cm, around the previous platform location) over swimming in the corresponding regions in the three other quadrants.

### Barnes maze

The Barnes maze circular holeboard (Ugo Basile) was 100 cm in diameter with 20 holes (5 cm in diameter). An escape box was placed under one of the holes and the box was filled halfway with bedding material plus two food pellets and the bedding was mixed after each mouse. The circular board was divided into 20 equal sectors and an inner area 15 cm from the edge of the maze (diameter 70 cm) while the outer area of each sector was used as a goal zone in the analysis carried out by Ethovision XT13 video-tracking.

#### Adaptation

Before the experiment started, the animals were familiarized with the goal box and trained to enter the goal box voluntarily (surrounding the hole with a large cylinder to confine the mouse close to the goal, allow some time to explore and placed there manually if the mouse did not enter the box in 3 min).

#### Days 1–3: training

Three trials per day (until escape into the goal box or max duration 180 s) with inter-trial interval for at least 1 h. Before the trial started, the mouse was placed in the non-transparent cylinder in the center of the arena. After 15–20 s, the cylinder was removed and the animal was free to explore the arena. If the animal entered the goal box, it was kept there for 10–15 s and then the box was removed with the mouse. If the mouse did not find or enter the box in 180 s, it was placed close to goal box so that it could still escape into the box after release. If the mouse still did not enter the box, it was placed there by hand.

#### Days 4–5

On day 4, a probe trial (90 s) was carried out without the escape box. Thereafter, learning a new place (opposite to the original) was carried out in three trials during day 4 and two trials on day 5, followed by a second probe trial on day 5.

### Contextual fear conditioning

The experiments were carried out in a computer-controlled fear conditioning system (TSE). Training was performed in a transparent acrylic arena (23 × 23 × 35 cm) within a constantly illuminated (~100 lx) conditioning chamber with a loudspeaker providing a constant, white background noise (68 dB). The mice were allowed to explore the arena for 3 min. Thereafter, a foot shock (0.6 mA, 2 s, constant current) was administered twice with a 30-s interval. The trial ended 30 s after the second foot shock. Contextual memory was tested 24 h after the training. The mice were returned to the conditioning arena and the total time of freezing (defined as an absence of any movements for more than 3 s) was measured by infrared light beams (scanned continuously with a frequency of 10 Hz) for 3 min.

### Grid hanging

This test was used to determine static muscle force. The mouse was placed on a 20 × 25 cm wire grid (grid unit 1 × 1 cm) that was carefully placed upside down as the lid of a 24 × 35.5 × 24 cm transparent plastic cage. The time of fall off until a cutoff time of 300 s was measured with a stopwatch. The test was repeated three times with a 10-min interval between the trials and the best result was recorded.

### Elevated plus maze

The elevated plus maze test is a widely used measure of anxiety. The maze consisted of four arms (30 × 5 cm) radiating from a central platform (5 × 5 cm) 40 cm above the floor. Two of the arms had no walls on any side (open) and two had a 14-cm high wall on all sides except at the center of the platform (closed). The maze was made of black plastic, but the arms were covered with a white plastic mat to provide a contrast with the mouse for the video image. The mouse was placed on the central platform and video-recorded for 5 min. The number of transitions between the arms and the time spent in the open and closed arms were calculated and the percentage of the total time spent in the open arms was analyzed. If the mouse made fewer than four arm visits, it was excluded from the analysis.

### Statistical analysis

All experiments were repeated at least twice, unless otherwise stated. Data are presented as mean ± s.e.m. An unpaired two-tailed Student’s *t*-test was used for comparisons between two groups. A one-way ANOVA with Tukey’s or Dunnett’s multiple comparison post hoc test was used for comparisons between three or more groups with one factor. A two-way ANOVA with Tukey’s or Dunnett’s multiple comparison post hoc test was used for comparison of multiple factors. A two-way ANOVA for repeated measures, three-way ANOVA for repeated measures or three-way repeated measures mixed-effects model with Tukey’s or Dunnett’s or Sidak’s multiple comparison post hoc test were used for comparison of multiple factors at various time points. Statistical analyses were performed using GraphPad Prism for MacOSX (v.9.0, GraphPad Software). Differences were considered statistically significant at *P* < 0.05.

### Reporting summary

Further information on research design is available in the Nature Portfolio Reporting Summary linked to this article.

## Extended Data

## Supplementary Material

Reporting summary

Source Data Extended Data Fig. 1

Source Data Extended Data Fig. 2

Source Data Extended Data Fig. 3

Source Data Extended Data Fig. 5

Source Data Extended Data Fig. 6

Source Data Extended Data Fig. 7

Source Data Extended Data Fig. 8

Source Data Extended Data Fig. 9

Source Data Extended Data Fig. 10

Source Data Fig. 1

Source Data Fig. 1

Source Data Fig. 2 Excel

Source Data Fig. 4

Source Data Fig. 4

Source Data Fig. 5

Source Data Fig. 6

Source Data Fig. 7

Supplementary Table 1

Supplementary Table 2

Supplementary Information

## Data Availability

All data supporting the findings in this study are included in the main article and Supplementary Information. Source data detailing the values used in quantifications mentioned in the text and figures are available in the online version of this paper. Source data are provided with this paper.

## References

[1] De Strooper B, Karran E (2016). The cellular phase of Alzheimer’s disease. Cell.

[2] Heneka MT (2015). Neuroinflammation in Alzheimer’s disease. Lancet Neurol.

[3] Jack CR (2013). Tracking pathophysiological processes in Alzheimer’s disease: an updated hypothetical model of dynamic biomarkers. Lancet Neurol.

[4] Hardy J, Selkoe DJ (2002). The amyloid hypothesis of Alzheimer’s disease: progress and problems on the road to therapeutics. Science.

[5] Zlokovic BV (2011). Neurovascular pathways to neurodegeneration in Alzheimer’s disease and other disorders. Nat Rev Neurosci.

[6] Kisler K, Nelson AR, Montagne A, Zlokovic BV (2017). Cerebral blood flow regulation and neurovascular dysfunction in Alzheimer disease. Nat Rev Neurosci.

[7] Karran E, Mercken M, De Strooper B (2011). The amyloid cascade hypothesis for Alzheimer’s disease: an appraisal for the development of therapeutics. Nat Rev Drug Discov.

[8] Bateman RJ (2006). Human amyloid-β synthesis and clearance rates as measured in cerebrospinal fluid in vivo. Nat Med.

[9] Mawuenyega KG (2010). Decreased clearance of CNS β-amyloid in Alzheimer’s disease. Science.

[10] Tarasoff-Conway JM (2015). Clearance systems in the brain-implications for Alzheimer disease. Nat Rev Neurol.

[11] Zlokovic BV (1995). Cerebrovascular permeability to peptides: manipulations of transport systems at the blood–brain barrier. Pharm Res.

[12] Iliff JJ (2012). A paravascular pathway facilitates CSF flow through the brain parenchyma and the clearance of interstitial solutes, including amyloid β. Sci Transl Med.

[13] Carare RO (2008). Solutes, but not cells, drain from the brain parenchyma along basement membranes of capillaries and arteries: significance for cerebral amyloid angiopathy and neuroimmunology. Neuropathol Appl Neurobiol.

[14] Key A, Retzius G (1876). Studien in der Anatomie des Nervensystems und des Bindegewebes.

[15] Schwalbe G (1869). Die Arachnoidalraum ein Lymphraum und sein Zusammenhang mit den Perichorioidalraum. [The arachnoidal space as a lymphatic space with connection to the perichoroidal compartment]. Zbl Med Wiss.

[16] Weed LH (1914). Studies on cerebro-spinal fluid. no. III: the pathways of escape from the subarachnoid spaces with particular reference to the arachnoid villi. J Med Res.

[17] Jacob L (2019). Anatomy and function of the vertebral column lymphatic network in mice. Nat Commun.

[18] Ma Q, Ineichen BV, Detmar M, Proulx ST (2017). Outflow of cerebrospinal fluid is predominantly through lymphatic vessels and is reduced in aged mice. Nat Commun.

[19] Kida S, Weller RO, Zhang ET, Phillips MJ, Iannotti F (1995). Anatomical pathways for lymphatic drainage of the brain and their pathological significance. Neuropathol Appl Neurobiol.

[20] Aspelund A (2015). A dural lymphatic vascular system that drains brain interstitial fluid and macromolecules. J Exp Med.

[21] Louveau A (2015). Structural and functional features of central nervous system lymphatic vessels. Nature.

[22] Antila S (2017). Development and plasticity of meningeal lymphatic vessels. J Exp Med.

[23] Helakari H (2022). Human NREM sleep promotes brain-wide vasomotor and respiratory pulsations. J Neurosci.

[24] Proulx ST (2021). Cerebrospinal fluid outflow: a review of the historical and contemporary evidence for arachnoid villi, perineural routes, and dural lymphatics. Cell Mol Life Sci.

[25] de Leon MJ (2017). Cerebrospinal fluid clearance in Alzheimer disease measured with dynamic PET. J Nucl Med.

[26] Pappolla M (2014). Evidence for lymphatic Aβ clearance in Alzheimer’s transgenic mice. Neurobiol Dis.

[27] Nauen DW, Troncoso JC (2022). Amyloid-β is present in human lymph nodes and greatly enriched in those of the cervical region. Alzheimers Dement.

[28] Ahn JH (2019). Meningeal lymphatic vessels at the skull base drain cerebrospinal fluid. Nature.

[29] Louveau A (2018). CNS lymphatic drainage and neuroinflammation are regulated by meningeal lymphatic vasculature. Nat Neurosci.

[30] Patel TK (2019). Dural lymphatics regulate clearance of extracellular tau from the CNS. Mol Neurodegener.

[31] Da Mesquita S (2018). Functional aspects of meningeal lymphatics in ageing and Alzheimer’s disease. Nature.

[32] Da Mesquita S (2021). Meningeal lymphatics affect microglia responses and anti-Aβ immunotherapy. Nature.

[33] Wang L (2019). Deep cervical lymph node ligation aggravates AD-like pathology of APP/PS1 mice. Brain Pathol.

[34] Wen YR, Yang JH, Wang X, Yao ZB (2018). Induced dural lymphangiogenesis facilities soluble amyloid-β clearance from brain in a transgenic mouse model of Alzheimer’s disease. Neural Regen Res.

[35] Leppapuska IM (2022). Phase 1 Lymfactin(R) study: 24-month efficacy and safety results of combined adenoviral VEGF-C and lymph node transfer treatment for upper extremity lymphedema. J Plast Reconstr Aesthet Surg.

[36] Makinen T (2001). Inhibition of lymphangiogenesis with resulting lymphedema in transgenic mice expressing soluble VEGF receptor-3. Nat Med.

[37] Jankowsky JL (2004). Mutant presenilins specifically elevate the levels of the 42 residue β-amyloid peptide in vivo: evidence for augmentation of a 42-specific λ secretase. Hum Mol Genet.

[38] Garcia-Alloza M (2006). Characterization of amyloid deposition in the APPswe/PS1dE9 mouse model of Alzheimer disease. Neurobiol Dis.

[39] Minkeviciene R (2008). Age-related decrease in stimulated glutamate release and vesicular glutamate transporters in APP/PS1 transgenic and wild-type mice. J Neurochem.

[40] Ghoneim FM (2015). Protective effect of chronic caffeine intake on gene expression of brain derived neurotrophic factor signaling and the immunoreactivity of glial fibrillary acidic protein and Ki-67 in Alzheimer’s disease. Int J Clin Exp Pathol.

[41] Oakley H (2006). Intraneuronal β-amyloid aggregates, neurodegeneration, and neuron loss in transgenic mice with five familial Alzheimer’s disease mutations: potential factors in amyloid plaque formation. J Neurosci.

[42] Jawhar S, Trawicka A, Jenneckens C, Bayer TA, Wirths O (2012). Motor deficits, neuron loss, and reduced anxiety coinciding with axonal degeneration and intraneuronal Aβ aggregation in the 5XFAD mouse model of Alzheimer’s disease. Neurobiol Aging.

[43] Li Z (2023). Blockade of VEGFR3 signaling leads to functional impairment of dural lymphatic vessels without affecting autoimmune neuroinflammation. Sci Immunol.

[44] Rustenhoven J (2021). Functional characterization of the dural sinuses as a neuroimmune interface. Cell.

[45] Song E (2020). VEGF-C-driven lymphatic drainage enables immunosurveillance of brain tumours. Nature.

[46] Tammela T (2011). Photodynamic ablation of lymphatic vessels and intralymphatic cancer cells prevents metastasis. Sci Transl Med.

[47] Lynch DH (1989). Systemic immunosuppression induced by photodynamic therapy (PDT) is adoptively transferred by macrophages. Photochem Photobiol.

[48] Mittal M, Siddiqui MR, Tran K, Reddy SP, Malik AB (2014). Reactive oxygen species in inflammation and tissue injury. Antioxid Redox Signal.

[49] Elmets CA, Bowen KD (1986). Immunological suppression in mice treated with hematoporphyrin derivative photoradiation. Cancer Res.

[50] Jolles CJ, Ott MJ, Straight RC, Lynch DH (1988). Systemic immunosuppression induced by peritoneal photodynamic therapy. Am J Obstet Gynecol.

[51] Canti G, Franco P, Marelli O, Ricci L, Nicolin A (1984). Hematoporphyrin derivative rescue from toxicity caused by chemotherapy or radiation in a murine leukemia model (L1210). Cancer Res.

[52] Choi D (2022). Piezo1-regulated mechanotransduction controls flow-activated lymphatic expansion. Circ Res.

[53] Ma L (2023). Skull progenitor cell-driven meningeal lymphatic restoration improves neurocognitive functions in craniosynostosis. Cell Stem Cell.

[54] Li X (2022). Meningeal lymphatic vessels mediate neurotropic viral drainage from the central nervous system. Nat Neurosci.

[55] Bolte AC (2020). Meningeal lymphatic dysfunction exacerbates traumatic brain injury pathogenesis. Nat Commun.

[56] Boisserand LSB VEGF-C prophylaxis favors lymphatic drainage and modulates neuroinflammation in a stroke model. J Exp Med.

[57] Tsai HH (2022). Functional Investigation of meningeal lymphatic system in experimental intracerebral hemorrhage. Stroke.

[58] Han J (2015). Vascular endothelial growth factor receptor 3 controls neural stem cell activation in mice and humans. Cell Rep.

[59] Yoon JH (2024). Nasopharyngeal lymphatic plexus is a hub for cerebrospinal fluid drainage. Nature.

[60] Breslin JW (2007). Vascular endothelial growth factor-C stimulates the lymphatic pump by a VEGF receptor-3-dependent mechanism. Am J Physiol Heart Circ Physiol.

[61] Madisen L (2015). Transgenic mice for intersectional targeting of neural sensors and effectors with high specificity and performance. Neuron.

[62] Ehling M, Adams S, Benedito R, Adams RH (2013). Notch controls retinal blood vessel maturation and quiescence. Development.

[63] Bry M (2010). Vascular endothelial growth factor-B acts as a coronary growth factor in transgenic rats without inducing angiogenesis, vascular leak, or inflammation. Circulation.

[64] He Y (2005). Vascular endothelial cell growth factor receptor 3-mediated activation of lymphatic endothelium is crucial for tumor cell entry and spread via lymphatic vessels. Cancer Res.

[65] Fang S (2016). Critical requirement of VEGF-C in transition to fetal erythropoiesis. Blood.

[66] Alitalo AK (2013). VEGF-C and VEGF-D blockade inhibits inflammatory skin carcinogenesis. Cancer Res.

[67] Anisimov A (2009). Activated forms of VEGF-C and VEGF-D provide improved vascular function in skeletal muscle. Circ Res.

[68] Paxinos G, Franklin KBJ (2001). The Mouse Brain in Stereotaxic Coordinates.

[69] Avants BB, Tustison N Advanced normalization tools (ANTS).

[70] Montoya-Zegarra JA (2019). AutoTube: a novel software for the automated morphometric analysis of vascular networks in tissues. Angiogenesis.

[71] Deacon RM (2006). Assessing nest building in mice. Nat Protoc.

