# Supplementary material for: Sustained meningeal lymphatic vessel atrophy or expansion does not alter Alzheimer’s disease-related amyloid pathology
Source: Nat Cardiovasc Res. Author manuscript; Available in PMC 2024 Jul 31. (PMC7616318; doi:10.1038/s44161-024-00445-9)
Supplement: Reporting summary [file EMS196559-supplement-Reporting_summary.pdf]

nature portfolio

Corresponding author(s): Salli Antila, Kari Alitalo

Last updated by author(s): 24.01.2024

Reporting Summary

Nature Portfolio wishes to improve the reproducibility of the work that we publish. This form provides structure for consistency and transparency in reporting. For further information on Nature Portfolio policies, see our Editorial Policies and the Editorial Policy Checklist.

Statistics

For all statistical analyses, confirm that the following items are present in the figure legend, table legend, main text, or Methods section.

n/a	Confirmed	
☐	☒	The exact sample size (*n*) for each experimental group/condition, given as a discrete number and unit of measurement
☐	☒	A statement on whether measurements were taken from distinct samples or whether the same sample was measured repeatedly
☐	☒	The statistical test(s) used AND whether they are one- or two-sided<br>*Only common tests should be described solely by name; describe more complex techniques in the Methods section.*
☐	☒	A description of all covariates tested
☐	☒	A description of any assumptions or corrections, such as tests of normality and adjustment for multiple comparisons
☐	☒	A full description of the statistical parameters including central tendency (e.g. means) or other basic estimates (e.g. regression coefficient) AND variation (e.g. standard deviation) or associated estimates of uncertainty (e.g. confidence intervals)
☐	☒	For null hypothesis testing, the test statistic (e.g. *F*, *t*, *r*) with confidence intervals, effect sizes, degrees of freedom and *P* value noted<br>*Give P values as exact values whenever suitable.*
☒	☐	For Bayesian analysis, information on the choice of priors and Markov chain Monte Carlo settings
☒	☐	For hierarchical and complex designs, identification of the appropriate level for tests and full reporting of outcomes
☒	☐	Estimates of effect sizes (e.g. Cohen's *d*, Pearson's *r*), indicating how they were calculated

Our web collection on statistics for biologists contains articles on many of the points above.

Software and code

Policy information about availability of computer code

Data collection

The following software were used to collect the data in this study:
- EnSight Multimode Plate Reader (Perkin Elmer)
- AxioZoom.V16 fluorescence stereo zoom microscope (Carl Zeiss) with OptiMOS sCMOS camera (QImaging) and ZEN 2012 software (Carl Zeiss)
- LSM 780 confocal microscope (Carl Zeiss) with ZEN 2011 software (Carl Zeiss)
- Pannoramic 250 Flash III fluorescence slide scanner (3DHISTECH)
- NanoZoomer XR slide scanner (Hamamatsu Photonics, Japan)
- Activity Monitor, V5.8 (MedAssociates)
- Ethovision XT13 videotracking (Noldus, The Netherlands)
- Computer-controlled fear conditioning system (TSE, Bad Homburg, Germany)

More detailed data collection information is reported in Online Methods.

Data analysis

The following software were used to analyze the data in this study:
- ImageJ software, V2.0.0-rc-69/1.52n (National Institutes of Health)
- GraphPad Prism for MacOSX V9.0 (GraphPad Software, San Diego California, USA)
- CaseViewer 2.4 software (3DHISTECH)
- NDP.view 2 software (Hamamatsu Photonics)

More detailed data analysis information is reported in Online Methods.

For manuscripts utilizing custom algorithms or software that are central to the research but not yet described in published literature, software must be made available to editors and reviewers. We strongly encourage code deposition in a community repository (e.g. GitHub). See the Nature Portfolio guidelines for submitting code & software for further information.

Data

Policy information about availability of data

All manuscripts must include a data availability statement. This statement should provide the following information, where applicable:
- Accession codes, unique identifiers, or web links for publicly available datasets
- A description of any restrictions on data availability
- For clinical datasets or third party data, please ensure that the statement adheres to our policy

All data supporting the findings in this study are included in the main article and associated files. Source data excel file detailing the values and statistical tests used in quantifications mentioned in the text and figures are available in the online version of this paper. All datasets generated and/or analyzed during the current study are available from the corresponding authors on reasonable request.

Research involving human participants, their data, or biological material

Policy information about studies with human participants or human data. See also policy information about sex, gender (identity/presentation), and sexual orientation and race, ethnicity and racism.

Reporting on sex and gender: NA

Reporting on race, ethnicity, or other socially relevant groupings: NA

Population characteristics: NA

Recruitment: NA

Ethics oversight: NA

Note that full information on the approval of the study protocol must also be provided in the manuscript.

Field-specific reporting

Please select the one below that is the best fit for your research. If you are not sure, read the appropriate sections before making your selection.

☒ Life sciences ☐ Behavioural & social sciences ☐ Ecological, evolutionary & environmental sciences

For a reference copy of the document with all sections, see nature.com/documents/nr-reporting-summary-flat.pdf

Life sciences study design

All studies must disclose on these points even when the disclosure is negative.

Sample size

Sample size for each experiment and indication of what the visualized datapoints represent are stated in every Figure legend. No statistical tests were used to pre-determine sample size. Instead, the number of required samples for each experiment was chosen based on previous experiments and comparative studies in literature.

Data exclusions

In Figure 1n, one value from APdE9-Ctrl group was identified as an outlier based on ROUT method (Q = 0.1%) and removed from the analysis. The removed value did not affect the result (significant treatment effect) and is present in the source data excel file in the online version of this paper.

In Extended Data Figure 8t quantification, one value from 5xFAD-Ctrl group was identified as an outlier based on ROUT method (Q = 5%) and Grubbs' method (Alpha = 0.05) and values from this mouse were removed from the cortex analysis in panels t-v. The removed values did not affect the result (non-significant) and are present in the source data excel file in the online version of this paper.

Replication

Main experimental findings are representative of at least two independent experiments using littermate mice. The number of reliable reproductions for each experiment are stated in every Figure legend.

Randomization	After knowing the mouse genotype (WT, K14-sR3, APdE9, 5xFAD), littermate animals were allocated into treatment groups randomly so that every experimental set included similar number of mice from every experimental group.
Blinding	The investigators were blinded to experimental group allocation during data collection and analysis.

Reporting for specific materials, systems and methods

We require information from authors about some types of materials, experimental systems and methods used in many studies. Here, indicate whether each material, system or method listed is relevant to your study. If you are not sure if a list item applies to your research, read the appropriate section before selecting a response.

Materials & experimental systems

n/a	Involved in the study	
☐	☒	Antibodies
☒	☐	Eukaryotic cell lines
☒	☐	Palaeontology and archaeology
☐	☒	Animals and other organisms
☒	☐	Clinical data
☒	☐	Dual use research of concern
☒	☐	Plants

Methods

n/a	Involved in the study	
☒	☐	ChIP-seq
☒	☐	Flow cytometry
☐	☒	MRI-based neuroimaging

Antibodies

Antibodies used

Non-conjugated primary antibodies:
- goat anti-mouse podocalyxin (1:500, R&D Systems, AF1556, AB_354858)
- rat anti-mouse podocalyxin (1:500; R&D Systems, MAB1556)
- rat anti-mouse endomucin (1:200, Santa Cruz Biotechnology, clone V.7C7)
- rabbit anti-mouse Von Willebrand Factor (1:500, Dako, A0082)
- goat anti-RFP (1:500, Rockland, 200-101-379)
- goat anti-human PROX1 (1:200, R&D Systems, AF2727, AB_2170716)
- polyclonal rabbit anti-mouse LYVE1 (1:1000, produced inhouse)
- rat anti-mouse LYVE1 (1:300, R&D Systems, MAB2125, AB_2138528)
- polyclonal goat anti-mouse VEGFR3 (1:50, R&D Systems, AF743, AB_355563)
- Syrian hamster anti-mouse podoplanin (1:100, DSHB, 8.1.1-s, AB_531893)
- mouse anti-amyloid-β 1-16 clone WO-2 (1:40000 for floating sections and 1:1000 for slides, Merck Millipore, MABN10, AB_10561919)
- rabbit anti-amyloid-β 1-37/42 clone D54D2 (1:1000, Cell Signaling, 8243S, AB_2797642)
- rabbit anti-AQP4 (1:2000, AB3594, Merck Millipore/Sigma Aldrich)

Conjugated antibodies
- goat PE conjugated anti-human IgG (Thermo Fisher, PA1-86078, AB_933621)
- mouse anti-alpha-SMA Cy3 conjugate (1:500, Sigma-Aldrich, clone 1A4, C6198)
- rabbit anti-amyloid-β 1-37/42 Alexa Fluor 647 conjugate (Cell Signaling, 42284, clone D54D2, 1:500 for dura mater)

Validation

Antibodies were either obtained from indicated commercial vendors with ensured quality or have been previously produced, validated, and published. The antibodies are well described, and specific references can be found on the manufacturer's website, in the antibody registry (https://antibodyregistry.org/) or from the indicated references. All immunohistochemical experiments included known negative and positive internal staining controls to validate the specific antibody signals.

Animals and other research organisms

Policy information about studies involving animals; ARRIVE guidelines recommended for reporting animal research, and Sex and Gender in Research

Laboratory animals	All mouse strains used have been previously published and are reported in Online Methods under "Mice" section. All mice were maintained on a C57BL/6J background.
Wild animals	The study did not involve wild animals.
Reporting on sex	Both female and male mice were used in the studies. The sex of mice used in every experiment is reported in Figure legends.
Field-collected samples	The study did not involve samples collected from the field.
Ethics oversight	All animal experiments were approved by the Animal Experiment Board of Finland.

Note that full information on the approval of the study protocol must also be provided in the manuscript.

Magnetic resonance imaging

Experimental design

Design type	NA
Design specifications	NA
Behavioral performance measures	NA

Acquisition

Imaging type(s)	Structural imaging
Field strength	7T
Sequence & imaging parameters	3D multigradient echo, TR = 68 ms, TE = 2.73 ms, echo spacing 2.9 ms, echoes 13, flip angle 16°, matrix size 125 µm3.
Area of acquisition	Whole brain was included in the FOV

Diffusion MRI ☐ Used ☒ Not used

Preprocessing

Preprocessing software	The images were intensity bias field inhomogeneity corrected with N4BiasCorrection from Advanced normalization tools (http://stnava.github.io/ANTs/).
Normalization	The corrected images were thresholded within the brain so that the resulting mask included 1% of the highest intensities of non-zero voxels.
Normalization template	NA
Noise and artifact removal	No noise or artifact removal was performed.
Volume censoring	NA

Statistical modeling & inference

Model type and settings	NA
Effect(s) tested	Ventricle volume comparison was performed with two-way ANOVA with Tukey's multiple comparison post hoc test.

Specify type of analysis: ☐ Whole brain ☐ ROI-based ☐ Both

Statistic type for inference (See Eklund et al. 2016)	NA
Correction	NA

Models & analysis

n/a	Involved in the study
☒	☐ Functional and/or effective connectivity
☒	☐ Graph analysis
☒	☐ Multivariate modeling or predictive analysis
