# Supplementary figures and images for: Sustained meningeal lymphatic vessel atrophy or expansion does not alter Alzheimer’s disease-related amyloid pathology
Source: Nat Cardiovasc Res. Author manuscript; Available in PMC 2024 Jul 31. (PMC7616318; doi:10.1038/s44161-024-00445-9)

## Uncropped Western Blot images

### Main Figures:

Fig.1 (b) 6m

VEGFR-3 (1:1000, # AF743, R&D).

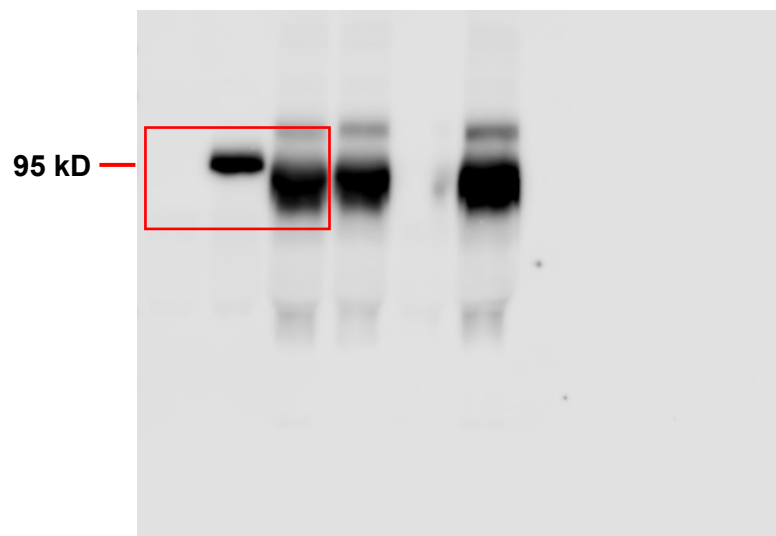

Fig.1 (b) 16m

VEGFR-3 (1:1000, # AF743, R&D).

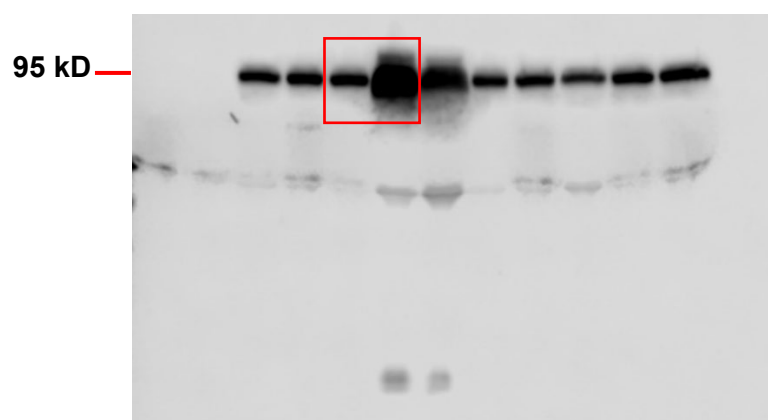

Supplement: Source Data Fig. 1 [file EMS196559-supplement-Source_Data_Fig__1.pdf]

**Fig.4 (b)**

**VEGFR-3 (1:1000, # AF743, R&D).**

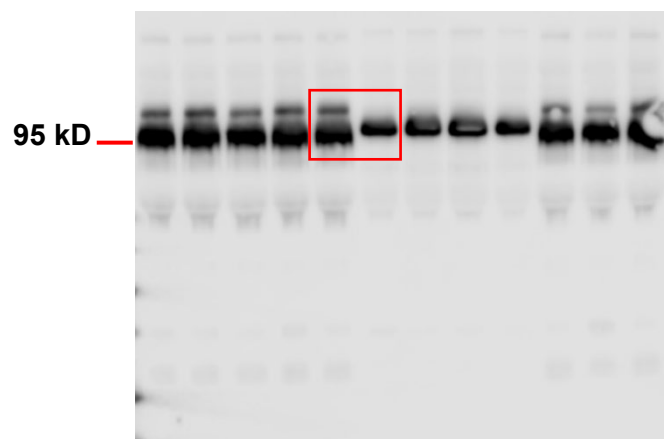

Supplement: Source Data Fig. 4 [file EMS196559-supplement-Source_Data_Fig__4.pdf]
